# The Autism Impact Measure (AIM): Meaningful Change Thresholds and Core Symptom Changes Over One Year from an Online Survey in the U.S.

**DOI:** 10.1007/s10803-022-05635-7

**Published:** 2022-07-04

**Authors:** Mariabeth Silkey, Gonzalo Durán-Pacheco, Michelle Johnson, Chuang Liu, Susanne Clinch, Kiely Law, Georg Loss

**Affiliations:** 1 F. Hoffmann-La Roche Ltd, Basel, Switzerland; 2 Roche Products Ltd, Welwyn Garden City, UK; 3 Kennedy Krieger Institute, Baltimore, MD USA; 4 Johns Hopkins University School of Medicine, Baltimore, MD USA; 5 Hexagon Place, 6 Falcon Way, Shire Park, Welwyn Garden City, AL7 1TW UK

**Keywords:** Autism spectrum disorder, Autism Impact Measure, Longitudinal data analysis, Minimal clinically important difference, Caregiver Global Impression of Change, Caregiver-reported anchors

## Abstract

Validated outcome measures with the capacity to reflect meaningful change are key to assessing potential interventions for autism spectrum disorder (ASD). We derive clinically meaningful change thresholds (MCTs) of the Autism Impact Measure (AIM) and identify factors associated with meaningful change. Baseline and 12-months follow-up survey of caregivers of 2,761 children with ASD aged 3–17 years from the U.S. Simons Foundation Powering Autism Research for Knowledge (SPARK) cohort were analyzed. Using caregiver-reported anchors for change, the 12-month change in estimated AIM MCT (95% confidence interval) for symptom improvement was –4.5 (–7.61, –1.37) points and 9.9 (5.12, 14.59) points for symptom deterioration. These anchor-based MCTs will facilitate future assessments of caregiver-reported change in AIM scores.

**Supplementary Information:**

The online version contains supplementary material available at 10.1007/s10803-022-05635-7.

## Introduction

Autism spectrum disorder (ASD) is a neurodevelopmental condition that is categorized by deficits in core symptoms of social communication, social interaction, and restricted, repetitive behaviors (Diagnostic and Statistical Manual of Mental Disorders (DSM-5), 2013). The expression of both core and associated symptoms is broad and varied, ranging from mild to severe impairment (Diagnostic and Statistical Manual of Mental Disorders (DSM-5), 2013). The diagnosis of ASD worldwide is increasing, either because of changes in diagnostic criteria, changes in primary diagnosis, an actual increase in incidence, or a combination of the above (Polyak et al., 2015). A variety of treatments and interventions are under development. For these to be evaluated, validated outcome measures with the ability to show meaningful changes in the core characteristics of ASD are required. The commonly used measures to assess core ASD symptoms were originally designed for screening or diagnostic purposes, and, thus, are not well-suited for measuring short-term improvement or deterioration (Ghosh et al., 2013; Grzadzinski et al., 2020; Kanne et al., 2014). Development of psychometrically validated outcome measures and calculating clinically meaningful thresholds are key priorities for autism research (Bolte & Diehl, 2013).

The Autism Impact Measure (AIM) is a caregiver-reported questionnaire designed to characterize core symptoms of ASD in individuals aged 3–18 years (Kanne et al., 2014; Mazurek et al., 2018). Although the U.S. Food and Drug Administration (FDA) recommends that the individual experiencing the symptoms report these themselves, a parent or caregiver may also report on observations of the individual’s experience, if that individual is a child or lacks cognitive insight (Food & Drug Administration, 2018). The AIM measures frequency of symptom occurrence and associated functional impact, which are both important for understanding symptom severity and permitting prioritization of interventions (Kanne et al., 2014; Mazurek et al., 2018). Key advantages of the AIM include that it has been designed for use as an outcome measure specifically for ASD, is less time-consuming than other interview-administered measures, and, importantly, it does not require trained personnel for administration. Therefore, the AIM could also be a suitable tool for real-world monitoring of pediatric ASD symptomology (Houghton et al., 2019). Cross-sectional studies have demonstrated that AIM has good test–retest reliability, cross-informant reliability, and convergent validity with other measures of ASD symptoms (Houghton et al., 2019; Kanne et al., 2014). Five symptom domains have been identified in the assessment of the AIM’s structural validity: repetitive behavior, atypical behavior, communication, social reciprocity, and peer interaction (Mazurek et al., 2018). Furthermore, the AIM has been used to detect symptom improvements after short-term interventions (Mazurek et al., 2020).

Clinical outcome measures used to evaluate the effectiveness of treatments must not only be sensitive to symptom changes but should also be able to demonstrate the clinical meaningfulness of those changes and, thus, of potential treatment effects. Interpreting score changes and the clinical meaningfulness of these changes can be done either at the group level, called the clinically important difference (CID), or at the individual level, called the within-person meaningful change threshold (MCT) (Food & Drug Administration, 2018). The within-person MCT is the magnitude of change in a clinical outcome assessment that needs to be observed in order to interpret whether there has been a meaningful improvement or, in some indications, deterioration (Coon & Cappelleri, 2016; Revicki et al., 2008). Within-person MCTs can be calculated using approaches such as anchor-based methods and distribution-based methods (Coon & Cappelleri, 2016; Food & Drug Administration, 2018; Revicki et al., 2008). An advantage of the former is that it incorporates the patient or caregiver voice using data from an external indicator (anchor), whereas distribution-based methods, typically used for cross-sectional data, consider the properties of the measure itself, such as 0.5 or 0.2 standard deviations of the measure of interest at baseline or the standard error of the measurement (Coon & Cappelleri, 2016; Fayers & Hays, 2014; Food & Drug Administration, 2018; Norman et al., 2003; Revicki et al., 2008). FDA guidance references that approaches to detect meaningful change at the individual level (within-person) are preferred using anchor-based approaches (typically, the use of Global Impression of Severity or Change scales), supplemented with distribution-based approaches for cross-sectional data (Food & Drug Administration, 2018). Anchor-based approaches depend upon the quality of the anchor; therefore, when calculating the clinically meaningful threshold of an outcome measure, triangulation of multiple methods for estimating MCTs, which includes the use of multiple independent anchors, should be assessed (Revicki et al., 2008). Even following such approaches, it is worth noting that MCTs of the same measure can vary by population and context. Distribution-based MCTs for the AIM have been previously estimated from cross-sectional data from children with ASD aged 3–17 years (Houghton et al., 2019). Additionally, Mazurek et al. (Mazurek et al., 2020) looked at short-term (typically 6 weeks) changes in AIM scores after specific interventions, and their correlations to other commonly used outcome measures including the Aberrant Behavior Checklist, the Social Responsiveness Scale-2, and the Vineland Adaptive Behavior Scales-2. The primary objective of our study was to calculate preliminary within-person MCTs for the AIM using anchor-based approaches over a 12-month interval, in order to aid the interpretation of the AIM in future studies.

## Methods

### Study Design

The data for this non-interventional, longitudinal study were derived from responses to two electronic caregiver surveys that were administered 12 months apart. The baseline cohort was sampled from the U.S.-wide Simons Foundation Powering Autism Research for Knowledge (SPARK) online research initiative (SPARK Consortium, 2018). Families participating in SPARK complete a battery of questionnaires to enter the cohort, and, thereafter, academic or industry researchers can recruit the same families into their studies. All data generated within SPARK are anonymized and made linkable by unique identifiers. Participant recruitment, online consent, and data collection were facilitated by SPARK; details of these methods and the results of the baseline assessment have been previously published (Houghton et al., 2019; Monz et al., 2019). At baseline, participants completed demographic queries and the AIM questionnaire between September 2017 and October 2017. The follow-up assessment between September 2018 and October 2018 included additional demographic queries, the AIM questionnaire, and two questionnaires not included in the baseline assessment: Caregiver-reported Global Impression of Change (CaGI-C) and Severity (CaGI-S) in Autism. The research protocol was approved by the Western Institutional Review Board (tracking number 20181254) and the study complied with the Guidelines for Good Pharmacoepidemiology (International Society for Pharmacoepidemiology, 2015).

Caregivers of individuals with ASD aged 3–17 years who participated in the baseline assessment and submitted an AIM questionnaire with ≤ 20% of missing items were eligible for the follow-up assessment. Eligible participants were primary caregivers for individuals with ASD for at least 12 months before the follow-up survey date. Email invitations to participate in the follow-up surveys were sent between September 12, 2018, and October 16, 2018; up to three reminders were sent to potential participants who did not respond to the initial invitation.

All respondents who provided complete information for the AIM score in both surveys and reported the CaGI-C impression of overall change (CaGI-C overall) at follow-up were included in the analyses.

### Procedures

The AIM survey (completed by the caregiver) consists of 41 items, each of which is answered twice on a 5-point Likert-type scale, once for frequency of a specific behavior of the dependent and once for impact of that behavior. Response options range from “never” to “always” for the frequency dimension and “not at all” to “severely” for the impact dimension. Three summary scores can be derived from the AIM: (1) total score, where all 41 items are summed across both dimensions (score ranging from 82–410); (2) frequency or impact dimension, where all items are summed for the individual dimension (score ranging from 41–205); (3) sums of the frequency and impact dimensions of questions corresponding to specific domains (repetitive behavior, communication, atypical behavior, social reciprocity, and peer interaction), which together represent 29 of the 41 items. The possible score ranges for each domain are 16–80 for repetitive behavior, 12–60 for communication, 12–60 for atypical behavior, 10–50 for social reciprocity, and 8–40 for peer interaction (Mazurek et al., 2018). Higher domain and higher total scores indicate greater symptom severity (Houghton et al., 2019; Mazurek et al., 2018). Likewise, lower AIM scores indicate less severe symptomatology. AIM score change was defined as the difference between the AIM score at the follow-up and baseline surveys. AIM score percent change was defined as the AIM score difference as a percentage of the baseline value.

The CaGI-C Autism questionnaire, an adaptation of the Clinician Global Impressions Scale for Improvement (Busner & Targum, 2007), consists of four items (communication, social interaction, everyday activities, and overall ASD) rated on a 7-point Likert-type scale ranging from scores 1 (“very much improved”) to 7 (“very much worse”). The primary anchor was change in overall ASD during the previous 12 months (CaGI-C overall). Secondary, domain-specific anchors were the dependents’ change in communication in the past 12 months (CaGI-C communication) and the dependents’ change in social interaction in the past 12 months (CaGI-C social interaction).

The CaGI-S Autism questionnaire, an adaptation of the Clinician Global Impressions Scale for Severity (Busner & Targum, 2007), asks how much difficulty a child has experienced in three areas (communication, social interaction, and everyday activities) over a 2-week recall and is rated on a 5-point Likert-type scale ranging from scores 1 (“no difficulty”) to 5 (“extreme difficulty”). The final item asks how severe the child’s overall ASD has been ranging from 1 (“not at all severe”) to 5 (“extremely severe”) with a score 3 (“moderately severe”) at the center.

### Data Analysis

Categorical variables were described using counts and proportions. Continuous variables were described using means and standard deviation (SD), medians, and interquartile range (IQR). Group differences for categorical and continuous variables were tested using the chi-squared, Fisher’s exact, or Student’s t-test as appropriate. All thresholds in the main and sensitivity analyses were calculated separately for absolute change and change relative to baseline values. Multivariable analyses (95% confidence interval [CI]) were reported, where applicable. All analyses were conducted in the statistical software R (R Core Team [2019] version 3.6.3). Details regarding missing data and sample size considerations appear in the supplementary methods.

#### Primary Anchor-Based Clinically Meaningful Change Thresholds

The primary anchor applied in this study was the dependent’s change in overall ASD over the previous 12 months as reported on the CaGI-C item regarding overall ASD change. In order to anchor longitudinal (12-month) changes in the AIM, only the CaGI-C was used, seeing as baseline data is needed to use the CaGI-S as an anchor, which was not available. Both improvement and deterioration were assessed since, a priori, we could not assume that the AIM score response to change was linear and symmetrical. An initial evaluation of the relation of the AIM change score and anchors was conducted graphically. Spearman’s correlations between AIM change scores and the CaGI-C anchors were calculated. AIM within-person MCTs (Coon & Cappelleri, 2016; Mamolo et al., 2015) were derived by comparing change in (or percent change from baseline of) total AIM score with CaGI-C overall. Specifically, a 7-point CaGI-C ordinal-scaled anchor was re-categorized into three main groups to identify subjects who experienced any improvement, i.e., those with CaGI-C score of 3 (“minimally improved”) or numerically lower, those who experienced any deterioration, i.e., CaGI-C score of 5 (“minimally worse”) or numerically higher, and those who stayed the same, i.e., CaGI-C score of 4 (“no change”). Linear models and one degree of freedom contrasts were used to estimate the MCTs as the difference in change scores from those who improved versus those with “no change” and for those who worsened versus those with “no change”; i.e., changes in the CaGI-C, coded as a binary variable (improved vs same or worsened), were modeled by within-person change in AIM score. A responder was defined as a subject with an AIM change exceeding the anchor-based MCT in either direction, i.e., MCTs were reported separately for responders of improvement and for responders of deterioration.

#### Secondary Anchor-Based Clinically Meaningful Change Thresholds

To assess the most appropriate anchor for the symptom class AIM domains, we first applied the primary anchor to each domain as detailed above. In addition, we applied different secondary anchors from symptom class-specific questions on the CaGI-C and CaGI-S. Within-person MCTs for secondary domain-specific anchors were calculated as described above with the following modifications. Change scores on the AIM communication domain were compared with the communication anchor. Similarly, change scores on both the AIM social reciprocity domain and the peer interaction domain were compared with the social interaction anchor.

#### Factors Associated with Changes in AIM

A multivariable linear regression was applied to identify those factors associated with changes in total AIM score. Further, a multivariable logistic regression of demographic factors associated with response status was conducted. Model covariates included total AIM score at baseline, demographic variables including age, sex, and race, whether the child was verbal, whether the child had eloped or strayed in the previous 12 months, the presence of mental health comorbidity, whether the child had been hospitalized or seen in the emergency room (ER) in the previous 12 months for mental health care, child suspended or expelled from school, years since ASD diagnosis, child takes (any) drug for ASD, caretaker age, household income, household size, U.S. region, and whether the child lived in an urban or rural setting.

#### Intelligence Quotient (IQ) Subsample

Within-person MCTs were stratified by cognitive impairment (IQ $\le$ 70, IQ > 70) (Maenner et al., 2020) among children with at least one IQ result reported in either the baseline or follow-up survey. For the approximately 70 children whose IQ score was not reported at baseline, the IQ reported at the follow-up was taken. Differences between the IQ subsample and total analysis population regarding key demographic variables were examined.

### Sensitivity Analyses

#### Equidistance

The main analysis allowed that the AIM score might be unequally sensitive to improvement or deterioration. In order to test the impact of this assumption, we re-ran the primary analysis with the equidistant constraint in place, that is, scores ≤ 3 were grouped and coded numerically as –1 (improved), scores equal to 4 were coded 0 (the same), and scores ≥ 5 were coded 1 (worse). A linear model was applied to estimate a single MCT as the change in AIM corresponding to one category unit of the integer CaGI-C anchor.

#### AIM Change Stability

The AIM score scale range of 340 points (80–420) is more granular than the 7-level CaGI-C scale used as the anchor for this study. To assess the stability and possible bias of the AIM scores, summary statistics of the AIM scores of those participants who reported “no change” during the year are presented.

#### Effect Size Considerations

In order to confirm that greater anchor changes relate to greater AIM score changes, a re-analysis of the data with the expectation of larger effect size was conducted. The CaGI-C scale was re-categorized with a greater distance between the improved and deteriorated classes: scores ≤ 2 were coded as “much improved,” scores ≥ 3 to ≤ 5 coded as the same, and scores ≥ 6 were coded as “much deteriorated.” All analyses were repeated for both the primary and secondary anchors.

#### Empirical, Within-Person MCTs Derived Using the Receiver Operating Characteristic

Commonly used in medical decision making, bioinformatics, data mining and machine learning, evaluating biomarker performances, or comparing scoring methods, the receiver operating characteristic (ROC) plot displays the performance of a binary classification method with continuous or discrete ordinal output. It shows the sensitivity (the proportion of correctly classified positive observations) and specificity (the proportion of correctly classified negative observations) as the output threshold is moved over the range of all possible values (Robin et al., 2011). Following Bara (Bara et al., 2018) and Farrar (Farrar et al., 2001), robust model-independent estimates of the within-person MCTs were derived by summarizing the number of correctly identified responders for a given AIM change in a ROC curve. The optimal empirically derived MCT was identified as the threshold corresponding to the highest area under the ROC curve using the pROC R package (Robin et al., 2011).

## Results

### Study Population and Descriptive Analysis

**Fig. 1:**
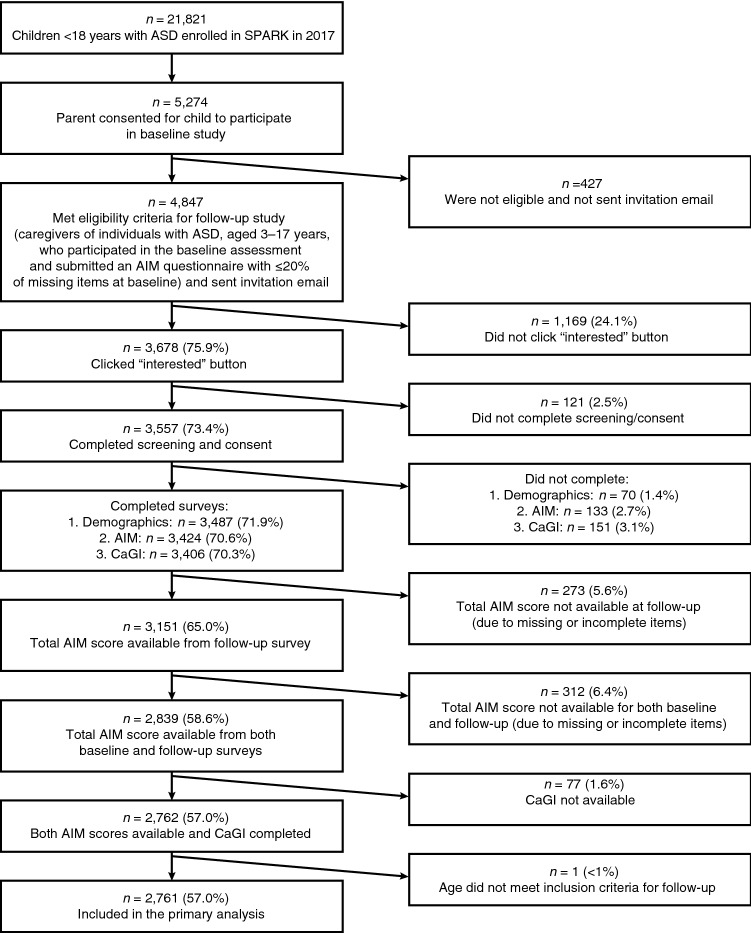
Participant flow diagram for follow-up study. ^a^Eligibility criteria: caregivers of individuals with ASD, aged 3–17 years, who participated in the baseline assessment and submitted an AIM questionnaire with ≤ 20% of missing items at baseline; Parentheses show % of total participants sent the invitation email. *AIM* Autism Impact Measure, *ASD* autism spectrum disease, *CaGI* Caregiver-reported Global Impression surveys, *SPARK* Simons Foundation Powering Autism Research for Knowledge

Of the caregivers who participated in the baseline study, 4847 (91.9%) were eligible for follow-up and were sent an invitation email, and reminders, where applicable (Fig. 1). Of these, 75.9% registered interest in participating in the follow-up surveys with 73.4% completing screening and consent forms. A total of 3424 (70.6%) and 3406 (70.3%) caregivers completed the AIM and CaGI-C surveys, respectively. The primary analysis population consisted of 2761 (57.0%) individuals with ASD under the age of 18 years whose caregiver reported information sufficient to calculate total AIM scores from both the baseline and follow-up surveys and who responded to the CaGI-C question regarding overall change in ASD.

At follow-up, children with ASD had a median age of 9.0 years (IQR 7.0–13.0 years), 79.7% were male, and 48.1% had been diagnosed with another mental health comorbidity (Table 1). See Table S1 for additional baseline characteristics and demographics. Caregivers had a median age of 39.0 years (IQR 34.0–44.0 years), 98.8% were parents of the dependents with ASD and 86.9% stated that their child was verbal (Table 1). Each of the geographic regions of the U.S. and income categories were well-represented. Overall, the distributions of key demographic variables of children with ASD and their caregivers in the primary analysis population at follow-up were generally comparable to those observed in the baseline study (Houghton et al., 2019) and to those observed among participants who completed demographics at follow-up but who were excluded from the primary analysis, except for a higher percentage of missing IQ scores (those who reported taking an IQ test but did not report the score) in the excluded group. Of the children with an IQ score available (*n* = 1,078), 74.0% had an IQ score > 70.

**Table 1:** Baseline characteristics of analysis population (caregiver and child with ASD)

Characteristic	Overall	Participants reporting IQ
*N*	2761	1078
Caregivers		
Median age, years (IQR)	39.0 (34.0, 44.0)	41.0 (36.0, 45.0)
Relation to child		
Parent	2723 (98.8)	1064 (99.0)
Legal guardian	25 (0.9)	8 (0.8)
Other	8 (0.3)	3 (0.3)
Missing	5	3
Children with ASD (age 4–17)		
Median age, years (IQR)	9.0 (7.0, 13.0)	11.0 (9.0, 14.0)
Gender		
Male	2186 (79.7)	844 (78.9)
Female	556 (20.3)	226 (21.1)
Missing	19	8
IQ score^a^		
≤ 70	280 (24.5)	280 (26.0)
71–99	366 (32.0)	366 (34.0)
≥ 100	432 (37.7)	432 (40.1)
Took test but did not report score	67 (5.9)	0
Did not report having taken an IQ test	1616	0
Caregiver reported child is verbal		
Yes	2399 (86.9)	1017 (94.3)
No	361 (13.1)	61 (5.7)
Prefer not to answer	1	0
Mental health comorbidities (any)		
Yes	1300 (48.1)	656 (60.9)
No	1402 (51.9)	406 (37.7)
Don’t know/missing	59	16
Ethnicity		
White/Non-Hispanic	1892 (68.5)	785 (72.8)
White/Hispanic	281 (10.2)	86 (8.0)
Non-white/Non-Hispanic	440 (15.9)	162 (15.0)
Non-white/Hispanic	148 (5.4)	45 (4.2)
U.S. region		
West	685 (24.8)	227 (21.1)
Midwest	626 (23.0)	247 (22.9)
Northeast	430 (15.6)	190 (17.6)
South	1016 (36.8)	413 (38.4)
Unknown	4	1

^a^Of the 1145 caregivers who reported that an IQ test had been given to their child, 1078 reported the actual IQ score result

Numbers indicate *n* (%) unless otherwise specified

*ASD* autism spectrum disorder, *IQ* intelligence quotient, *IQR* interquartile range

### AIM Scores

At the follow-up assessment, the mean (SD) total AIM score was 208.9 (54.2), total AIM frequency score was 115.4 (26.7), and total AIM impact score was 93.5 (30.5; Table 2). Overall, total AIM and domain scores were, on average, lower at follow-up compared with baseline (Table 2).

**Table 2:** AIM scores at baseline and follow-up

	Baseline value, mean (SD)	Follow-up value, mean (SD)
Total AIM	221.36 (53.75)	208.91 (54.24)
AIM Frequency	120.16 (26.17)	115.39 (26.69)
AIM Impact	101.21 (30.44)	93.53 (30.48)
AIM Repetitive Behavior domain	41.59 (13.71)	39.85 (13.60)
AIM Communication domain	30.69 (11.80)	27.91 (11.32)
AIM Atypical Behavior domain	34.83 (9.95)	32.69 (9.93)
AIM Social Reciprocity domain	27.14 (7.21)	25.71 (7.38)
AIM Peer Interaction domain	22.83 (7.06)	21.56 (7.05)

*AIM* Autism Impact Measure, *SD* standard deviation

**Fig. 2:**
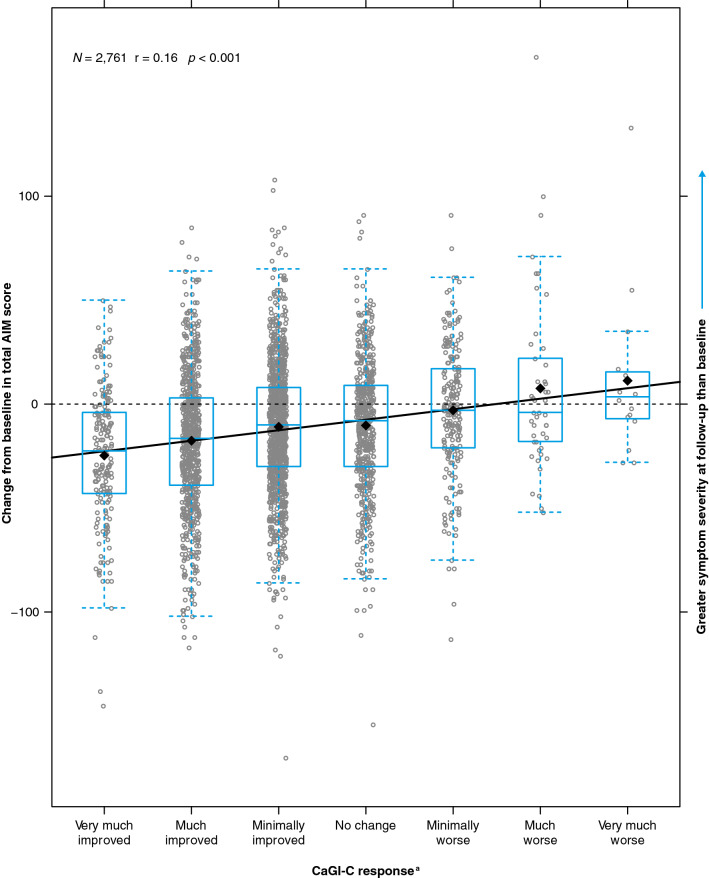
Change in total AIM score versus the primary anchor (overall caregiver impression of change). ^a^Response to question “Please indicate how much change your child has experienced between 12 months ago and today in his/her overall autism.” Horizontal bars show medians, boxes show interquartile ranges, whiskers show Tukey intervals. *AIM* Autism Impact Measure, *CaGI-C* Caregiver-reported Global Impression of Change survey

Correlations for the AIM change scores against the CaGI-C overall ranged from 0.08 < r_Spearman_ < 0.18, with all being significant at the 1% level (*p* < 0.001; Figs. 2 and S1–S4). Calculated correlations for AIM domain scores at follow-up and specific questions of CaGI-S ranged from 0.45–0.63 (Fig. S5); the cross-sectional correlation between the AIM total score and the CaGI-S overall severity at follow-up was 0.631. Further, the change from baseline in AIM change scores (total or domain) against the primary (Figs. 2 and S1–S2) and secondary anchors (Figs. S3–S4) showed that the average AIM score changes were all negative (i.e., improved) for improvement categories on the anchor and AIM score changes were positive (i.e., worsening) for deterioration categories “much worse” or “very much worse” on the anchor. On the CaGI-C, 10.0% (*n* = 276) reported worsening of overall ASD.

### Primary and Secondary Anchor-Based Clinically Meaningful Change Thresholds

**Fig. 3:**
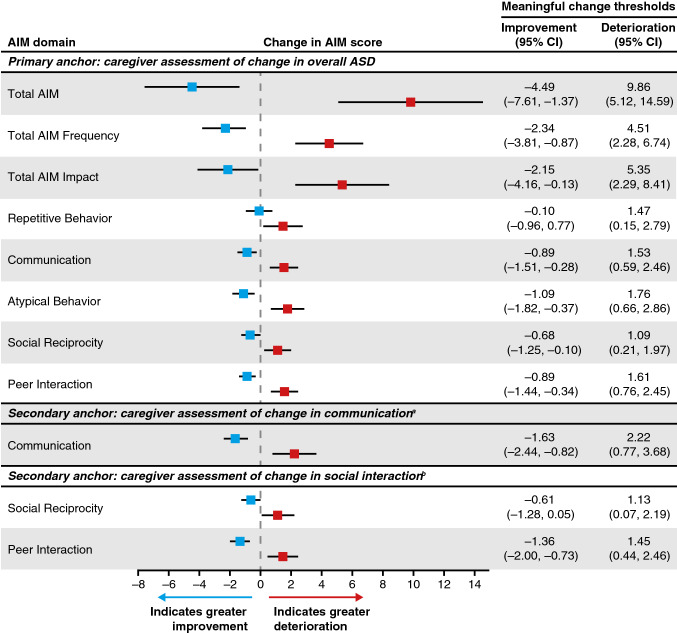
Primary and secondary anchor-based, within-person meaningful change thresholds for improvement and deterioration. ^a^*N* = 2761; ^b^*N* = 2760; AIM Autism Impact Measure, *ASD* autism spectrum disorder, *CI* confidence interval

The estimated AIM total score within-person MCTs (95% CI) over 12 months were –4.5 (–7.6, –1.4) for symptom improvement and 9.9 (5.1, 14.6) for symptom deterioration (Fig. 3). The step size for deterioration was nearly twice as large as that for improvement. The relative change in estimated AIM MCT (95% CI) over 12 months for symptom improvement was –2.6% (–4.0, –1.1) and 4.3% (2.1, 6.6) for symptom deterioration (Fig. S6).

### Primary and Secondary Anchor-Based Clinically Meaningful Percent Change Thresholds

Primary and secondary anchors can be compared using the percent change thresholds. Relative change estimates of improvement in the AIM social reciprocity domain anchored against the CaGI-C overall and CaGI-C social interaction assessments were consistent, –2.5% (–4.8, –0.2) and –2.1% (–4.7, 0.6), respectively, as were the relative change estimates for improvement in the AIM peer interaction domain anchored against the CaGI-C overall and CaGI-C social interaction assessments, –3.4% (–6.3, –0.6) and –4.6% (–7.8, –1.3), respectively (Fig. S6). However, the relative change estimate of improvement in the AIM communication domain anchored against the CaGI-C communication assessment was almost twice that for anchored against the CaGI-C overall, –4.6% (–7.2, –1.9) and –2.9% (–4.9, –0.9), respectively (Fig. S6). Likewise, MCTs for deterioration based on secondary anchors were slightly larger than those derived against the primary anchor. Specifically, communication 5.8 vs. 9.7 (overall anchor versus communication anchor), social reciprocity 4.0 vs. 4.6 (overall anchor versus social interaction anchor), and peer interaction 7.2 vs. 8.3 (overall anchor versus social interaction anchor) (Fig. S6).

### Multivariable Analysis of AIM Change

Factors associated with AIM score change are described in Table S2 and factors associated with MCT responses for improvement and deterioration in Tables S3–4. Higher total AIM scores at baseline and whether a child was verbal were strongly associated with a marked reduction of AIM scores over 12 months (improvement). Whether a child had eloped in the previous year was associated with deterioration (*p* < 0.001; Table S2). Likewise, higher total AIM scores at baseline, and whether a child was verbal were strongly associated with higher odds of MCT response for improvement while children who had eloped during the previous year was associated with increased odds of MCT response for deterioration (Tables S3–S4). Children from families with incomes > U.S.$50,000 or those who preferred not to answer had a lower risk of MCT response for deterioration compared with families with incomes < U.S.$50,000.

MCTs were separately summarized for a subset of children with IQ reported (280 children with IQ ≤ 70 and 789 children with IQ > 70; Table S5). Multiple regression results obtained from this population were similar to that of the overall study sample, with the exception that income no longer was associated with AIM change score (Table S6). However, children with IQ ≤ 70 had a significantly lower reduction in AIM scores over 12 months compared with children with IQ > 70 (*p* = 0.001).

### Sensitivity Analyses

Generally, the improvement MCTs were substantially smaller than respective deterioration MCTs, i.e., estimates were not equidistant. Additional modeling that assumed equidistance produced estimates with magnitude halfway between the two non-equidistant measurements for the primary and secondary anchors for all AIM outcomes (Table S7).

A median change score for those children whose caregivers reported no change severity overall tended towards improvement, for example the total AIM score decreased by a median (IQR) of 8 (–30, 9) points (Table S8).

MCTs derived for the “much improved”/“much deteriorated” definition of change did not scale linearly with the MCTs calculated in the main analysis, with most being around two to three times larger (Figs. S7–S8). The change in estimated total AIM MCT (95% CI) over 12 months for symptom “much improvement” was –9.3 (–11.9, –6.7) and 18.3 (10.1, 26.5) for “much deterioration” (Fig. S7).

MCTs for total AIM change scores derived from model-independent ROC analysis (Tables S9–S10) were similar to those derived from the primary results methodology for deterioration (10.5 and 9.9, respectively) versus the threshold obtained from equidistant anchor-based estimates (6.5; Table S7). For improvement, the ROC method produced a threshold for total AIM change score of higher magnitude than the equidistant and primary anchor-based estimates (–7.5 and –6.5 vs. –4.5).

## Discussion

In order for an outcome measure to be used in a clinical trial setting as an endpoint, it is important to understand at what point an improvement or worsening is interpreted as clinically meaningful, commonly referred to as the within-person MCT (Food & Drug Administration, 2018; Grzadzinski et al., 2020; Rai et al., 2015). This study used Global Impression of Change anchors to estimate within-person MCTs of the AIM over 12 months. We found that, in relation to the overall caregiver perception of change, a reduction of 4.5 points or more in the AIM total score could be used as a threshold estimate for symptom improvement, whereas an increase of 9.9 points is the estimated threshold for symptom deterioration. Our study adds to the body of work looking to understand the meaningfulness of AIM score change over time, by examining changes in a real-world cohort over an intermediate timeframe. The AIM is a simple caregiver questionnaire designed to capture core symptoms of ASD in children that can be used to measure the effectiveness of new interventions (Mazurek et al., 2020). While estimates of meaningful change can be derived from cross-sectional data, especially when longitudinal data are absent, evaluating AIM scores over time is critical to providing a robust and comprehensive threshold estimate. Previous longitudinal studies of ASD outcome measures were limited to regional populations where outcome data were available, generally not conducted to determine minimally clinical effect sizes of a specific outcome measure (Chatham et al., 2018; Grzadzinski et al., 2020; Pickles et al., 2020).

Our results are drawn from a large sample (*N* = 2,761) with high between-subject variability, circumstances that translate into very small but nonetheless highly significant correlations between AIM score change and CaGI-C (r_Spearman_ ~ 0.10, *p* < 0.001). The overall pattern of median AIM score change and categories of CaGI-C is, however, indicative of a direct but variable relationship between AIM score change and CaGI-C. Change scores lay noticeably below zero for CaGI-C categories reporting ASD improvement, incrementally increasing up to categories denoting ASD deterioration (Figs. 2, S1–S4). The correlation effect size indicates that the relationship is not strongly linear, but neither would it be expected to be a priori. The median of the AIM change score for those children whose caregivers reported no change during the year was 8 points (IQR –30, 9), suggesting that the children as a group experienced an improvement in symptoms over the year, which did not translate to the less granular CaGI-C anchor.

The AIM total score thresholds calculated here are based on a real-world cohort, with typically heterogeneous treatment plans and life circumstances. Using a multivariable analysis, we identified factors in this cohort that were consistently associated with AIM score change and MCT response status, co-factors that were identified as contributing to caregiver strain by Durán-Pacheco in the same cohort (Durán-Pacheco et al., 2022). Higher ASD symptom severity, as indicated by higher baseline AIM score, was related to important reductions of AIM score over time, and therefore increased odds of MCT response for improvement and reduced odds of MCT response for deterioration. Consistent with other papers where the association between verbosity and ASD severity has been demonstrated (Mayo et al., 2013; Nevill et al., 2019), a child’s ability to speak was associated with a decrease of AIM scores over time, and thus to increased odds of being a responder for improvement. Conversely, a child straying from or eloping was related to a noticeable increase of AIM score and consequently to increased odds of experiencing a meaningful worsening. Our results are consistent with another study that has shown that severity is related to the potential for symptom improvement, with less severe cases having higher potential for long-term improvement (Mayo et al., 2013).

Our study relied on a single anchor to evaluate the potential of the AIM to capture change over intermediate time scales. In a recent study designed to assess the ability of the AIM to detect changes identified by multiple other measures of ASD, the AIM was administered repeatedly over 6-week intervals throughout the duration of various treatments, varying from two to four follow-up visits depending on the treatment (Mazurek et al., 2020). Multiple repeated measures allowed Mazurek to account for both within- and between-subject variability and found that the model estimates of slope were similar to those found in the current study. Mazurek found that the total AIM score and AIM domain scores, with the exception of repetitive behavior, were sensitive to change overall and as a function of different treatment conditions (Mazurek et al., 2020). We also found that the AIM repetitive behavior domain was less sensitive to change compared with the other domains, suggesting this concept may not be of primary consideration when caregivers respond to the global anchor, or that repetitive behaviors in the sample remained relatively stable.

Generally, we found that the magnitude of an improvement threshold was half that of the respective deterioration threshold. Sensitivity analysis that assumed equidistance (where improvement and deterioration were not modeled separately) corroborated this result; estimates yielded were of magnitudes halfway between those for improvement and deterioration MCTs. Any natural bias in the AIM score was explored by summarizing the change in AIM score for those children whose caregivers reported no change in overall ASD during the previous year. One would expect that these differences, in the event of no bias, or no overall growth, would average around 0. However, we found that change trended towards improvement. The improvement/deterioration asymmetry may be a symptom of reporter bias: parents may be more inclined to report an improvement or confirm the status quo rather than report a deterioration in their child’s ASD. Alternatively, it could be that the AIM is constructed to be more sensitive to improvement rather than to deterioration of ASD symptoms.

In order to put the estimates from our primary methodology into context, we performed a variety of sensitivity analyses. We explored the impact of a larger “effect size” and, in line with our hypothesis, the MCTs derived for “much improvement” versus “much deterioration” were larger than those derived for the primary analysis. These larger thresholds (in addition to the main thresholds) are valuable data to help inform the relevance of AIM results in future studies. Optimal thresholds derived by simultaneously maximizing the sensitivity and specificity of potential thresholds using the ROC further supported our results, by producing results in the same direction and of a similar magnitude as those derived in our main analysis (a decrease of 7.5 [4.5, 14.5] indicating improvement and an increase of 10.5 [4.5, 11.5] indicating deterioration).

In order to examine change thresholds among domains, the percent change thresholds are required, since the range of possible domain scores varies. Likewise, the percent change thresholds reported allow the utility of the primary and secondary anchors applied to AIM domain scores for communication, peer interaction, and social reciprocity to be compared. Further studies relating AIM to other ASD outcome scales over diverse time scales will be needed to explore these relationships in more detail.

Baseline characteristics and demographics of the cohort children with ASD in this study showed similarity to children with ASD in the 2009–2010 National Survey of Children with Special Health Care Needs (NS-CSHCN) (Data Resource Center for Child & Adolescent Health, 2018), indicating that this cohort is broadly representative of children with ASD in the U.S., with the exception of a higher level of education for caregivers in this study.

Designing a study to calibrate AIM score changes presents several challenges, but as Mazurek noted, the greatest challenge is that there is no *“established gold standard measure of true change in ASD symptoms within or across domains”* (Mazurek et al., 2020). Although the electronic surveys were designed to limit missing data, overall, around 16% of the sample data needed to be excluded due to missing or incomplete items on the AIM either at baseline or follow-up. The majority of the surveys that were excluded were missing responses to a single item on the AIM questionnaire. A consideration for future studies is to explore suitable methods to imputing missing items to avoid excluding data. Other limitations are those typical of retrospective data collection. We tried to reduce caregivers’ recall bias by restricting the recall period for the two administrations of AIM survey to the last 2 weeks. Most characteristics of the caregivers and dependents with ASD were collected at baseline, and we assumed that demographic characteristics were stable during the study period.

This study is the largest nation-wide study, to our knowledge, to assess mid-term changes in AIM scores. The digital questionnaire employed in the study minimized the extent of data that might be missing, increasing their utility. Furthermore, these data, which contribute to the expanding data on the SPARK cohort, are publicly available from the Simons Foundation for Future Research by the ASD research community.

## Considerations

- Although the cross-sectional correlations between the CaGI-S and the AIM were reasonable (> 0.3), the longitudinal correlations of the CaGI-C were suboptimal. One reason for this may be because of the real-world dataset that was used for these analyses. Seeing as participants were not collectively taking treatment that would be expected to improve symptoms, it was not expected for there to be substantial changes reported on the change anchor. To test the estimated MCTs calculated from this study, future work should include the CaGI-S and CaGI-C, alongside the AIM in an intervention setting, thus where symptom change would be expected.
- MCTs calculated can vary by study population and rely on the robustness of the methods used to derive them (Coon & Cappelleri, 2016; Revicki et al., 2008). For these reasons, threshold “triangulation” has been proposed, whereby the aggregate body of evidence from multiple methods of determining MCTs is used to support the establishment of a single threshold or a narrow range of values (Coon & Cappelleri, 2016). This typically includes the use of multiple approaches and/or anchors, as well as expert consensus on what a range of MCTs might look like.
- In this study, data were analyzed for all participants, without subgroups for demographics such as age. However, if appropriate, MCTs for subgroups may be useful, assuming such subgroups substantially differ in terms of their disorder characteristics. In such a context, age-range specific MCTs would be useful for interventional clinical trials where eligibility is restricted to certain age groups. To this end, future studies of the AIM should assess diverse timescales (beyond 12 months) and subpopulations.

## Conclusions

Among a large sample of children with ASD in the United States, anchor-based, within-person MCTs for 12-month changes in AIM scores have been estimated. Whilst we acknowledge future work is needed to confirm these findings, such as triangulation of multiple approaches, this analysis represents important work in order to interpret changes of a relatively new measure. The preliminary MCTs presented will be relevant for both intervention development and, once approved, post-marketing of interventions to address core symptoms of ASD.

## Supplementary Information

Below is the link to the electronic supplementary material.

Supplementary file1 (PDF 1075 kb)

## Data Availability

For up-to-date details on Roche's Global Policy on the Sharing of Clinical Information and how to request access to related clinical study documents, see here: https://go.roche.com/data_sharing. Request for the data underlying this publication requires a detailed, hypothesis-driven statistical analysis plan that is collaboratively developed by the requestor and company subject matter experts. Such requests should be directed to datarequest.autism@roche.com for consideration. Anonymized records for individual patients across more than one data source external to Roche cannot, and should not, be linked due to a potential increase in risk of patient re-identification.
