# Correction: The Autism Impact Measure (AIM): Meaningful Change Thresholds and Core Symptom Changes Over One Year from an Online Survey in the U.S.

**DOI:** 10.1007/s10803-022-05700-1

**Published:** 2022-07-28

**Authors:** Mariabeth Silkey, Gonzalo Durán-Pacheco, Michelle Johnson, Chuang Liu, Susanne Clinch, Kiely Law, Georg Loss

**Affiliations:** 1 F. Hoffmann-La Roche Ltd, Basel, Switzerland; 2 Roche Products Ltd, Welwyn Garden City, UK; 3 Kennedy Krieger Institute, Baltimore, MD USA; 4 Johns Hopkins University School of Medicine, Baltimore, MD USA; 5 Hexagon Place, 6 Falcon Way, Shire Park, Welwyn Garden City, AL7 1TW UK

## Correction to: Journal of Autism and Developmental Disorders 10.1007/s10803-022-05635-7

In original article the Figure 3, the social reciprocity meaningful change threshold for improvement (95% CI) of:

− 0.68 (− 1.25, − 0.01)

Instead should say:

− 0.68 (− 1.25, − 0.10)

The original article has been corrected.

