# Supplementary material for: The Autism Impact Measure (AIM): Meaningful Change Thresholds and Core Symptom Changes Over One Year from an Online Survey in the U.S
Source: J Autism Dev Disord. 2022 Jul 4;53(9):3422–34. doi: 10.1007/s10803-022-05635-7 (PMC10465376; doi:10.1007/s10803-022-05635-7)
Supplement: Supplementary file 1 — Supplementary file1 (PDF 1075 kb) [file 10803_2022_5635_MOESM1_ESM.pdf]

Supplementary appendix

Supplement to:

Autism Impact Measure: Meaningful Change Thresholds and Core Symptom Changes Over One Year from an Online Survey in the U.S.

Mariabeth Silkey, Gonzalo Durán-Pacheco, Michelle Johnson, Chuang Liu, Susanne Clinch, Kiely Law, and Georg Loss

Journal of Autism and Developmental Disorders

Corresponding author: Michelle Johnson, Roche Products Ltd.
E-mail: michelle.johnson.mj2@roche.com

Contents

Additional methods	Page 2
Tables S1–S10	Pages 3–14
Figures S1–S8	Pages 15–22

Additional Methods

Handling of missing data

The digital questionnaire was designed to minimize the extent of missing data by limiting the selection of answers using drop-down menus; including “I don’t know” and “I prefer not to answer” for sensitive questions. Skip pattern mechanisms were used in hierarchical questions, which automatically registered “not applicable” for skipped questions. Questions answered in the survey would automatically proceed to the next appropriate question. Partially answered sections of the survey were automatically saved. Respondents of incomplete questionnaires received up to three email reminders, including a link to the survey to complete their responses.

Sample size considerations

Of 4,966 participants in the baseline study with AIM sufficiently completed, a response rate of 70% was expected for the follow-up study, equating to an expected sample size of 3,476 participants. CIR is commonly estimated as one-fifth the standard deviation (Fayers & Hays, 2014). At baseline (Monz et al., 2019), a standard deviation of 54.1 on total AIM score was observed, leading to an estimated CIR threshold of 10.8 (=0.2*54.1).

References

Fayers, P. M., & Hays, R. D. (2014). Don't middle your MIDs: regression to the mean shrinks estimates of minimally important differences. *Quality of Life Research, 23*(1), 1–4. https://doi.org/10.1007/s11136-013-0443-4

Monz, B. U., Houghton, R., Law, K., & Loss, G. (2019). Treatment patterns in children with autism in the United States. *Autism Research, 12*(3), 517–526. https://doi.org/10.1002/aur.2070

Table S1 Additional baseline characteristics of the analysis population

Parameter	Overall (n=2,761)	Participants reporting IQ (n=1,078)	Overall vs. group reporting IQ score *p*-value
Caregiver & Household			
Marital status			0.4817[a]
Single and never married	184 (6.7)	66 (6.2)	
Married	2,021 (73.7)	794 (74.1)	
Living with partner	169 (6.2)	51 (4.8)	
Separated	77 (2.8)	34 (3.2)	
Divorced	274 (10.0)	116 (10.8)	
Widowed	19 (0.7)	10 (0.9)	
Prefer not to answer	17	7	
Employment			0.6430[a]
Work ≥35 hours per week	1,182 (42.9)	489 (45.5)	
Work <35 hours per week	506 (18.4)	196 (18.2)	
Full-time homemaker	827 (30.0)	301 (28.0)	
Retired	19 (0.7)	7 (0.7)	
Student	69 (2.5)	27 (2.5)	
Unemployed	82 (3.0)	24 (2.2)	
Other	69 (2.5)	31 (2.9)	
Prefer not to answer	7	3	
Total household income in USD			0.0124[a]
<$50,000	1,018 (38.4)	353 (34.0)	
$50,000–$99,999	900 (33.9)	352 (33.9)	
≥$100,000	735 (27.7)	333 (32.1)	
Prefer not to answer	107	40	
Missing	1	0	
Household size including caregiver and child with ASD			0.8106[a]
<4	789 (28.6)	319 (29.7)	
4.0	1,072 (38.9)	412 (38.3)	
≥5	896 (32.5)	344 (32.0)	
Prefer not to answer	4	3	
Child with ASD			
Age group, years			<0.0001[a]
4	149 (5.4)	21 (2.0)	
5–9	1,287 (46.7)	353 (32.8)	
10–14	918 (33.3)	483 (44.9)	
15–17	402 (14.6)	218 (20.3)	
Missing	5	3	
Child eloped			0.3359[a]
Yes	649 (23.6)	238 (22.1)	
No	2,104 (76.4)	837 (77.9)	
Don't know/Prefer not to answer	8	3	
Child hospitalized for mental health care			0.0789[a]
Yes	63 (2.3)	36 (3.4)	
No	2,687 (97.7)	1,035 (96.7)	
Missing	11	7	
Child seen in emergency room for mental health care			0.0394[a]
Yes	110 (4.0)	60 (5.8)	
No	2,646 (96.0)	1,015 (94.4)	
Missing	5	3	
Child suspended or expelled from school			0.0791[a]
Yes	161 (5.8)	80 (7.4)	
No	2,594 (94.2)	995 (92.6)	
Missing	6	3	
Child attended school with special education students only			0.0156[a]
Yes	2,206 (80.3)	901 (83.7)	
No	542 (19.7)	175 (16.3)	

Parameter	Overall (n=2,761)	Participants reporting IQ (n=1,078)	Overall vs. group reporting IQ score *p*-value
Missing/Don't know	13	2	
Of children who attended school with typically developing peers (n=2,206; n= 901), percent time attended class			0.0553[a]
>60%	1,288 (60.4)	563 (64.0)	
30%–60%	321 (15.1)	136 (15.4)	
<30%	523 (24.5)	180 (20.5)	
Don't know	73	22	
Missing	1	0	
Prescription medication (any)			<0.0001[a]
Yes	1,430 (51.9)	672 (62.6)	
No	1,324 (48.1)	401 (37.4)	
Don't know	1	0	
Missing	6	5	
Of those reported taking prescription medication (n =1,420; n= 672), medication was for ASD symptoms and behaviors			0.0574[a]
Yes	898 (64.0)	454 (68.4)	
No	505 (36.0)	210 (31.6)	
Don't know	24	7	
Missing	3	1	
IQ test ever given			
Yes	1,220 (51.5)	991 (93.2)	
No	1,147 (48.5)	72 (6.8)	
Don't know	393	15	
Missing	1	0	
Of children with IQ scores reported (n=1,078), reported baseline IQ score ≤70			
Yes	280	280 (26.0)	
No	798	798 (74.0)	
Metropolitan statistical area			0.6870[a]
Macropolitan	2,244 (87.3)	877 (86.7)	
Micropolitan	326 (12.7)	134 (13.3)	
Missing	191	67	
Time since diagnosis			<0.0001[b]
Mean (SD)	5.4 (3.5)	6.1 (3.8)	
Missing	7	4	
Total AIM score at baseline<br>Mean (SD)	221.4 (53.8)	217.6 (53.3)	0.0501[b]

[a]The chi-square test was used to compare the distribution of categorical variables between the subset of children for whom an IQ score was known, and the overall group

[b]Continuous variables between the two groups were compared using the t-test

Note. Numbers indicate n (%); percent denominators do not include counts of Missing/Don't know/Prefer not to answer.

Abbreviations: *AIM* Autism Impact Measure*, ASD* autism spectrum disorder, *IQ* intelligence quotient, *SD* standard deviation, *USD* United States Dollar

Table S2 Factors associated with AIM change scores

**Variable**	**Estimate (95% CI)**	***p*-value**
Intercept	–0.87 (–12.08, 10.34)	0.8794
Child with ASD		
Age (centered around mean)	–0.40 (–0.95, 0.16)	0.1597
Gender (reference female)		
Child is male	–1.51 (–4.44, 1.42)	0.3117
Total AIM score at baseline (centered and scaled)	–12.25 (–13.55, –10.95)	<0.0001
Race (reference Non-white/Hispanic)		
Non-white/Non-Hispanic	–1.28 (–7.20, 4.64)	0.6720
White/Hispanic	0.19 (–6.10, 6.48)	0.9531
White/Non-Hispanic	0.18 (–5.18, 5.54)	0.9475
Child is verbal	–12.13 (–15.88, –8.37)	<0.0001
Child has eloped or strayed	11.22 (8.27, 14.16)	<0.0001
Child has other (unspecified) mental health comorbidities	–0.89 (–3.63, 1.86)	0.5272
Child has been hospitalized for mental health care	–0.88 (–10.92, 9.16)	0.8635
Child has been seen in ER for mental health care	2.15 (–5.59, 9.89)	0.5863
Child suspended or expelled from school	–1.64 (–6.85, 3.58)	0.5387
Years since diagnosis	0.57 (0.06, 1.08)	0.0292
Child takes (any) drug for ASD	1.35 (–1.33, 4.03)	0.3247
Caregiver & Household		
Caregiver age	–0.06 (–0.26, 0.15)	0.5970
Household Income (reference <$50,000)		
$50,000–$99,999	–3.52 (–6.42, –0.62)	0.0176
≥$100,000	–1.88 (–5.16, 1.42)	0.2638
Prefer not to answer	–8.85 (–15.24, –2.47)	0.0066
Household size (reference size <4)		
Household size: 4	–1.05 (–4.00, 1.90)	0.4860
Household size: ≥5	1.65 (–1.42, 4.73)	0.2924
U.S. region (reference region West)		
South	–1.70 (–4.80, 1.39)	0.2804
Midwest	–2.72 (–6.20, 0.76)	0.1256
Northeast	–1.86 (–5.69, 1.96)	0.3401
Metropolitan service area (reference macropolitan)		
Micropolitan	–2.04 (–5.78, 1.71)	0.2858
Unknown	–0.61 (–5.33, 4.11)	0.7998

Note. All factors listed in the table were included in the multivariable model for AIM score change. *P*-values arrived at by applying the t-test to coefficient estimates.

Abbreviations: *AIM* Autism Impact Measure, *ASD* autism spectrum disorder, *CI* confidence interval, *ER* emergency room

Table S3 Factors associated with a primary anchor meaningful change threshold response for improvement on total AIM

**Variable**	**Odds ratio (95% CI)**	***p*-value**
Intercept	0.66 (0.31, 1.44)	0.2994
Child with ASD		
Age (centered around mean)	1.01 (0.98, 1.05)	0.4766
Child is male	1.08 (0.89, 1.32)	0.4331
Total AIM score at baseline (centered and scaled)	1.75 (1.59, 1.92)	<0.0001
Race (reference Non-white/Hispanic)		
Non-white/Non-Hispanic	1.07 (0.71, 1.62)	0.7307
White/Hispanic	0.99 (0.64, 1.53)	0.9799
White/Non-Hispanic	0.94 (0.65, 1.36)	0.7570
Child is verbal	2.19 (1.69, 2.83)	<0.0001
Child has eloped or strayed	0.59 (0.48, 0.72)	<0.0001
Child has other (unspecified) mental health comorbidities	1.06 (0.88, 1.28)	0.5619
Child has been hospitalized for mental health care	0.99 (0.49, 2.02)	0.9751
Child has been seen in ER for mental health care	1.09 (0.64, 1.91)	0.7512
Child suspended or expelled from school	1.11 (0.77, 1.60)	0.5775
Years since diagnosis	0.98 (0.94, 1.01)	0.1860
Child takes (any) drug for ASD	0.94 (0.78, 1.13)	0.5101
Caregiver & Household		
Caregiver age	1.00 (0.99, 1.01)	0.9186
Household Income (reference <$50,000)		
$50,000–$99,999	1.28 (1.04, 1.56)	0.0172
≥$100,000	1.16 (0.92, 1.45)	0.2060
Prefer not to answer	1.68 (1.07, 2.69)	0.0259
Household size (reference size <4)		
Household size: 4	1.05 (0.86, 1.29)	0.6439
Household size: ≥5	1.05 (0.85, 1.30)	0.6577
U.S. region (reference region West)		
South	1.18 (0.95, 1.46)	0.1263
Midwest	1.33 (1.05, 1.69)	0.0200
Northeast	1.13 (0.87, 1.47)	0.3503
Metropolitan service area (reference macropolitan)		
Micropolitan	0.97 (0.75, 1.26)	0.8245
Unknown	0.94 (0.68, 1.31)	0.7307

Note. All factors listed in the table were included in the multivariable model for clinically important responder for improvement. *P*-values arrived at by applying the t-test to coefficient estimates.

Abbreviations: *AIM* Autism Impact Measure, *ASD* autism spectrum disorder, *CI* confidence interval, *ER* emergency room

Table S4 Factors associated with a primary anchor meaningful change threshold response for deterioration on total AIM

Variable	Odds ratio (95% CI)	*p*-value
Intercept	0.73 (0.29, 1.79)	0.4858
Child with ASD		
Age (centered around mean)	0.94 (0.90, 0.99)	0.0100
Child is male	0.89 (0.71, 1.12)	0.3163
Total AIM score at baseline (centered and scaled)	0.53 (0.47, 0.59)	<0.0001
Race (reference Non-white/Hispanic)		
Non-white/Non-Hispanic	1.19 (0.74, 1.95)	0.4722
White/Hispanic	1.01 (0.61, 1.71)	0.9549
White/Non-Hispanic	1.19 (0.78, 1.87)	0.4344
Child is verbal	0.53 (0.40, 0.72)	<0.0001
Child has eloped or strayed	1.87 (1.49, 2.36)	<0.0001
Child has other (unspecified) mental health comorbidities	0.98 (0.79, 1.23)	0.8710
Child has been hospitalized for mental health care	0.97 (0.39, 2.32)	0.9475
Child has been seen in ER for mental health care	0.88 (0.43, 1.69)	0.7114
Child suspended or expelled from school	0.84 (0.53, 1.28)	0.4311
Years since diagnosis	1.05 (1.00, 1.09)	0.0395
Child takes (any) drug for ASD	1.06 (0.85, 1.32)	0.5968
Caregiver & Household		
Caregiver age	0.99 (0.97, 1.00)	0.1568
Household Income (reference <$50,000)		
$50,000–$99,999	0.75 (0.59, 0.95)	0.0166
≥$100,000	0.75 (0.58, 0.98)	0.0345
Prefer not to answer	0.37 (0.19, 0.68)	0.0026
Household size (reference size <4)		
Household size: 4	0.85 (0.67, 1.08)	0.1721
Household size: ≥5	0.91 (0.71, 1.16)	0.4532
U.S. region (reference region West)		
South	0.86 (0.68, 1.10)	0.2428
Midwest	0.77 (0.58, 1.02)	0.0650
Northeast	0.82 (0.61, 1.12)	0.2165
Metropolitan service area (reference macropolitan)		
Micropolitan	1.08 (0.80, 1.44)	0.6152
Unknown	0.96 (0.65, 1.40)	0.8403

Note. All factors listed in the table were included in the multivariable model for clinically important responder for deterioration. *P*-values arrived at by applying the t-test to coefficient estimates.

Abbreviations: *AIM* Autism Impact Measure, *ASD* autism spectrum disorder, *CI* confidence interval, *ER* emergency room

Table S5. Meaningful change thresholds for children with IQ data (change and percent change from baseline)

	Meaningful change threshold of improvement		Meaningful change threshold of deterioration	
**Parameter**	**Improvement threshold (95% CI)**	**% Improvement threshold (95% CI)**	**Deterioration threshold (95% CI)**	**% Deterioration threshold (95% CI)**
**Children without cognitive impairment**				
**Primary anchor: caregiver impression of change in overall ASD**				
Total AIM	–3.62 (–8.82, 1.58)	–2.50 (–5.00, 0.10)	10.92 (2.60, 19.25)	4.70 (0.61, 8.87)
AIM Frequency	–1.85 (–4.40, 0.69)	–2.00 (–4.50, 0.50)	7.21 (3.14, 11.29)	5.90 (1.87, 9.88)
AIM Impact	–1.77 (–5.06, 1.52)	–3.00 (–6.50, 0.50)	3.71 (–1.56, 8.97)	3.30 (–2.28, 8.88)
AIM Repetitive Behavior Domain	–0.18 (–1.66, 1.31)	–1.50 (–5.40, 2.40)	1.99 (–0.38, 4.36)	5.00 (–1.25, 11.20)
AIM Communication Domain	–0.35 (–1.32, 0.62)	–2.40 (–6.10, 1.30)	2.08 (0.53, 3.64)	6.70 (0.76, 12.70)
AIM Aberrant Behavior Domain	–1.40 (–2.60, –0.20)	–4.60 (–8.30, –0.90)	2.33 (0.41, 4.25)	6.60 (0.64, 12.59)
AIM Social Reciprocity Domain	–0.78 (–1.77, 0.21)	–2.20 (–6.60, 2.10)	0.92 (–0.66, 2.51)	3.10 (–3.81, 10.06)
AIM Peer Interaction Domain	–0.82 (–1.80, 0.17)	–3.00 (–8.00, 2.00)	0.87 (–0.71, 2.45)	2.50 (–5.52, 10.43)
**Secondary anchor: caregiver impression of change in communication**				
AIM Communication Domain	–0.72 (–2.02, 0.58)	–2.30 (–7.30, 2.60)	2.43 (0.12, 4.74)	10.30 (1.42, 19.15)
**Secondary anchor: caregiver impression of change social interaction**				
AIM Peer Interaction Domain	–0.54 (–1.75, 0.67)	–0.90 (–6.20, 4.40)	2.32 (0.46, 4.18)	9.00 (0.82, 17.14)
AIM Social Reciprocity Domain	–1.23 (–2.43, –0.02)	–2.60 (–8.70, 3.50)	1.55 (–0.30, 3.40)	6.10 (–3.28, 15.51)
**Children with cognitive impairment**				
**Primary anchor: caregiver impression of change in overall ASD**				
Total AIM	–8.00 (–17.99, 2.00)	10.90 (–2.31, 24.18)	–3.80 (–7.84, 0.20)	4.00 (–1.33, 9.32)
AIM Frequency	–2.60 (–6.93, 1.78)	3.60 (–2.13, 9.42)	–2.60 (–5.91, 0.69)	1.80 (–2.57, 6.19)
AIM Impact	–5.40 (–12.17, 1.33)	7.30 (–1.65, 16.23)	–4.80 (–10.78, 1.22)	7.20 (–0.71, 15.18)
AIM Repetitive Behavior Domain	0 (–2.63, 2.64)	2.20 (–1.28, 5.70)	–0.60 (–6.34, 5.18)	3.70 (–3.94, 11.33)
AIM Communication Domain	–0.70 (–2.69, 1.25)	2.30 (–0.30, 4.93)	–2.50 (–8.34, 3.31)	7.40 (–0.33, 15.12)
AIM Aberrant Behavior Domain	0.10 (–2.17, 2.41)	3.40 (0.35, 6.43)	–0.40 (–6.65, 5.86)	8.90 (0.66, 17.23)
AIM Social Reciprocity Domain	–1.40 (–3.17, 0.27)	1.50 (–0.79, 3.77)	–5.30 (–11.41, 0.90)	6.40 (–1.79, 14.52)
AIM Peer Interaction Domain	–1.10 (–2.75, 0.60)	3.10 (0.86, 5.30)	–3.40 (–10.43, 3.72)	14.10 (4.76, 23.51)
**Secondary anchor: caregiver impression of change in communication**				
AIM Communication Domain	–1.50 (–3.82, 0.91)	4.80 (1.11, 8.46)	–3.50 (–10.45, 3.53)	16.00 (5.10, 26.82)
**Secondary anchor: caregiver impression of change social interaction**				
AIM Peer Interaction Domain	–1.40 (–3.27, 0.38)	0.60 (–2.07, 3.20)	–5.60 (–12.08, 0.95)	4.60 (–4.79, 14.05)
AIM Social Reciprocity Domain	–2.90 (–4.62, –1.12)	1.50 (–1.00, 4.05)	–11.20 (–18.54, –3.79)	8.60 (–2.07, 19.25)

Note. Linear-model-based (one degree of freedom) contrasts of AIM change scores comparing: "Improvement" vs. "No change" and "Deterioration" vs. "No change."

"Improvement": CaGI-C overall ASD assessed as "minimally improved", "much improved", or "very much improved."

"Deterioration": CaGI-C overall ASD assessed as "minimally worse", "much worse", or "very much worse."

"No change": CaGI-C overall ASD assessed as "no change."

% Improvement and % Deterioration is % change relative to baseline value.

Abbreviations: *AIM* Autism Impact Measure, *ASD* autism spectrum disorder, *CaGI-C* Caregiver-reported Global Impression of Change survey, *CI* confidence interval, *IQ* intelligence quotient

Table S6 Factors associated with AIM change scores for children with IQ data

Variable	Estimate	*p*-value
Intercept	–27.63 (–46.84, –8.42)	0.0049
Child with ASD		
Age (centered around mean)	–0.55 (–1.37, 0.28)	0.1939
Child is male	0.50 (–3.97, 4.96)	0.8276
Child IQ <70	9.20 (4.57, 13.83)	0.0001
Total AIM score at baseline (centered and scaled)	–13.39 (–15.48, –11.29)	<0.0001
Race (reference Non-white/Hispanic)		
Non-white/Non-Hispanic	1.34 (–8.88, 11.55)	0.7972
White/Hispanic	1.00 (–10.06, 12.06)	0.8591
White/Non-Hispanic	5.95 (–3.41, 15.31)	0.2122
Child is verbal	–12.27 (–20.82, –3.73)	0.0049
Child has eloped or strayed	11.06 (6.30, 15.81)	<0.0001
Child has other (unspecified) mental health comorbidities	0.17 (–3.99, 4.34)	0.9345
Child has been hospitalized for mental health care	–5.04 (–19.40, 9.33)	0.4917
Child has been seen in ER for mental health care	6.46 (–4.78, 17.70)	0.2600
Child suspended or expelled from school	–0.87 (–8.03, 6.28)	0.8105
Years since diagnosis	0.56 (–0.13, 1.25)	0.1121
Child takes (any) drug for ASD	1.09 (–3.07, 5.25)	0.6073
Caregiver & Household		
Caregiver age	0.27 (–0.05, 0.59)	0.1032
Household Income (reference <$50,000)		
$50,000–$99,999	–3.12 (–7.71, 1.47)	0.1827
≥$100,000	–4.50 (–9.56, 0.57)	0.0821
Prefer not to answer	–9.04 (–19.00, 0.93)	0.0754
Household size (reference size <4)		
Household size: 4	0.73 (–3.87, 5.33)	0.7556
Household size: ≥5	4.49 (–0.31, 9.28)	0.0667
U.S. region (reference region West)		
South	3.09 (–1.96, 8.13)	0.2302
Midwest	–1.95 (–7.56, 3.66)	0.4951
Northeast	1.23 (–4.67, 7.12)	0.6833
Metropolitan service area (reference macropolitan)		
Micropolitan	–0.11 (–5.77, 5.55)	0.9698
Unknown	0.60 (–6.87, 8.07)	0.8752

Note. All factors listed in the table were included in the multivariable model for AIM score change for children with IQ data. *P*-values arrived at by applying the t-test to coefficient estimates.

Abbreviations: *AIM* Autism Impact Measure, *ASD* autism spectrum disorder, *IQ* intelligence quotient

Table S7 Sensitivity analysis: Meaningful change thresholds estimated assuming equidistance anchors

Parameter	Estimate vs. anchor (95% CI) for improvement (−) or deterioration (+)[a]	R^2 from model	*p*-value	Spearman's rho (Parameter vs. CaGI anchor)
**Anchor: caregiver impression of overall change in ASD**				
Total AIM	6.50 (4.65, 8.35)	0.017	<0.0001	0.160
AIM Frequency	3.04 (2.17, 3.91)	0.018	<0.0001	0.160
AIM Impact	3.46 (2.26, 4.65)	0.011	<0.0001	0.129
AIM Repetitive Behavior Domain	0.64 (0.12, 1.15)	0.002	0.0214	0.076
AIM Communication Domain	1.00 (0.64, 1.36)	0.013	<0.0001	0.133
AIM Aberrant Behavior Domain	1.43 (1.01, 1.86)	0.014	<0.0001	0.124
AIM Social Reciprocity Domain	0.81 (0.47, 1.15)	0.008	<0.0001	0.109
AIM Peer Interaction Domain	1.15 (0.82, 1.48)	0.017	<0.0001	0.135
**Anchor: caregiver impression of change in communication**				
AIM Communication Domain	1.82 (1.30, 2.33)	0.017	<0.0001	0.161
**Anchor: caregiver impression of change in social interaction**				
AIM Social Reciprocity Domain	0.79 (0.40, 1.18)	0.006	<0.0001	0.111
AIM Peer Interaction Domain	1.39 (1.02, 1.77)	0.019	<0.0001	0.176

[a]The equidistant sensitivity analysis assumes that the improvement and deterioration anchors are of equal magnitude; the value of the improvement estimate is negative, and the value of the deterioration estimate is positive.

Note. The magnitude of the CIR threshold estimates derived in this analysis falls between the magnitude of the Improvement and Deterioration CIR threshold estimates in the primary analysis.

Abbreviations: *AIM* Autism Impact Measure, *ASD* autism spectrum disorder, *CI* confidence interval

Table S8 Variability of AIM score change for individuals identified by their caregiver as having no overall change in ASD (CaGI-C) at follow-up

**Parameter**	**Median change from baseline (IQR)**	**Mean change from baseline (SD)**
**Anchor: caregiver impression of overall change in ASD** (n=527)		
Total AIM	–8 (–30, 9)	–10.3 (32.1)
AIM Frequency	–4 (–12, 6)	–3.6 (14.2)
AIM Impact	–5 (–20, 6)	–6.7 (21.2)
AIM Repetitive Behavior Domain	–1 (–7, 4)	–1.8 (9.6)
AIM Communication Domain	–2 (–6, 1)	–2.3 (5.9)
AIM Aberrant Behavior Domain	–2 (–6, 3)	–1.5 (7.5)
AIM Social Reciprocity Domain	–1 (–5, 3)	–1.1 (6.0)
AIM Peer Interaction Domain	–1 (–4, 3)	–0.8 (5.5)
**Anchor: caregiver impression of change in communication** (n=271)		
AIM Communication Domain	–1 (–5, 2)	–1.5 (5.79)
**Anchor: caregiver impression of change in social interaction** (n=375)		
AIM Social Reciprocity Domain	–1 (–5, 3)	–1.0 (5.96)
AIM Peer Interaction Domain	0 (–3, 3)	–0.3 (5.45)

Note. The median change from baseline during the year for all subjects for whom the caregivers reported no overall change in ASD is slightly negative.

Abbreviations: *AIM* Autism Impact Measure, *ASD* autism spectrum disorder, *CaGI-C* Caregiver-reported Global Impression of Change survey, *IQR* interquartile range, *SD* standard deviation

Table S9 Sensitivity analysis: anchor-based meaningful change thresholds for improvement based on ROC analysis

	Improvement change				Improvement % change			
**Parameter**	**Threshold estimate (95% CI)[a]**	**AUC**	**Sensitivity**	**Specificity**	**Threshold estimate (95% CI)[a]**	**AUC**	**Sensitivity**	**Specificity**
**Primary anchor: caregiver impression of change in overall ASD**								
Total AIM	−7.50 (−14.50, −4.50)	0.57	0.59	0.53	−4.41 (−7.79, −2.41)	0.58	0.56	0.56
AIM Frequency	−3.50 (−4.50, −2.50)	0.57	0.56	0.54	−0.78 (−2.99, −2.53)	0.58	0.53	0.60
AIM Impact	−5.50 (−7.50, −3.50)	0.56	0.56	0.54	−6.46 (−8.51, −5.50)	0.57	0.56	0.57
AIM Repetitive Behavior Domain	−0.50 (−2.50, −0.50)	0.52	0.56	0.48	−2.90 (−8.91, −2.00)	0.53	0.54	0.51
AIM Communication Domain	−2.50 (−3.50, −1.50)	0.55	0.50	0.56	−5.83 (−11.81 −4.60)	0.56	0.58	0.52
AIM Aberrant Behavior Domain	−0.50 (−1.50, −0.50)	0.56	0.62	0.49	−2.41 (−7.22, −2.02)	0.57	0.61	0.51
AIM Social Reciprocity Domain	−0.50 (−2.50, −0.50)	0.55	0.58	0.49	−2.74 (−8.54, −2.60)	0.55	0.58	0.50
AIM Peer Interaction Domain	−1.50 (−1.50, −0.50)	0.57	0.51	0.61	−5.63 (−8.17, −3.51)	0.57	0.53	0.60
**Secondary anchor: caregiver impression of change in communication**								
AIM Communication Domain	−1.50 (−2.50, −1.50)	0.59	0.57	0.56	−5.06 (−4.21, −10.47)	0.59	0.59	0.57
**Secondary anchor: caregiver impression of change in social interaction**								
AIM Peer Interaction Domain	−1.50 (−1.50, −0.50)	0.59	0.51	0.66	−4.12 (−3.92, −5.97)	0.55	0.53	0.55
AIM Social Reciprocity Domain	−1.50 (−1.50, 0.50)	0.46	0.50	0.44	−4.65 (−8.76, −2.67)	0.59	0.55	0.64

[a]Empirical confidence interval derived from 5,000 bootstrap samples. Intervals are not bias corrected.

Abbreviations: *AIM* Autism Impact Measure, *ASD* autism spectrum disorder, *AUC* area under the curve, *CI* confidence interval, *ROC* receiver operator characteristic

Table S10 Anchor-based meaningful change thresholds for deterioration based on ROC analysis

	Deterioration change				Deterioration % change			
**Parameter**	**Threshold estimate (95% CI)[a]**	**AUC**	**Sensitivity**	**Specificity**	**Threshold estimate (95% CI)[a]**	**AUC**	**Sensitivity**	**Specificity**
**Primary anchor: caregiver impression of change in overall ASD**								
Total AIM	10.50 (4.50, 11.5)	0.61	0.65	0.53	4.08 (2.04, 5.49)	0.62	0.65	0.56
AIM Frequency	2.50 (0.50, 8.50)	0.61	0.61	0.57	1.71 (0.72, 7.39)	0.62	0.61	0.59
AIM Impact	5.50 (1.50, 7.50)	0.59	0.58	0.55	5.95 (3.93, 8.50)	0.60	0.63	0.55
AIM Repetitive Behavior Domain	–1.50 (–2.50, 2.50)	0.54	0.42	0.66	7.85 (3.31, 9.20)	0.56	0.68	0.43
AIM Communication Domain	1.50 (–0.50, 1.50)	0.58	0.57	0.57	4.49 (3.39, 7.62)	0.59	0.56	0.59
AIM Aberrant Behavior Domain	0.50 (–0.50, 0.50)	0.60	0.58	0.60	2.15 (0.94, 6.09)	0.61	0.59	0.60
AIM Social Reciprocity Domain	0.50 (–0.50, 2.50)	0.57	0.53	0.57	3.18 (1.11, 8.76)	0.57	0.55	0.56
AIM Peer Interaction Domain	0.50 (–0.50, –1.50)	0.61	0.58	0.56	4.08 (3.51, 8.17)	0.60	0.63	0.54
**Secondary anchor: caregiver impression of change in communication**								
AIM Communication Domain	1.50 (0.50, –1.50)	0.58	0.57	0.57	4.48 (3.39, 7.62)	0.59	0.56	0.59
**Secondary anchor: caregiver impression of change in social interaction**								
AIM Peer Interaction Domain	0.50 (–1.50, 1.50)	0.61	0.58	0.56	3.18 (–3 .51, 8.17)	0.67	0.55	0.56
AIM Social Reciprocity Domain	0.50 (–2.50, 0.50)	0.57	0.53	0.57	4.08 (2.67, 8.76)	0.60	0.63	0.54

[a]Empirical confidence interval derived from 5,000 bootstrap samples. Intervals are not bias corrected.

Abbreviations: *AIM* Autism Impact Measure, *ASD* autism spectrum disorder, *AUC* area under the curve, *CI* confidence interval, *ROC* receiver operator characteristic

Fig. S1 Change in AIM frequency score (a), impact score (b), and repetitive behavior domain score (c) versus the primary anchor (overall caregiver impression of change)

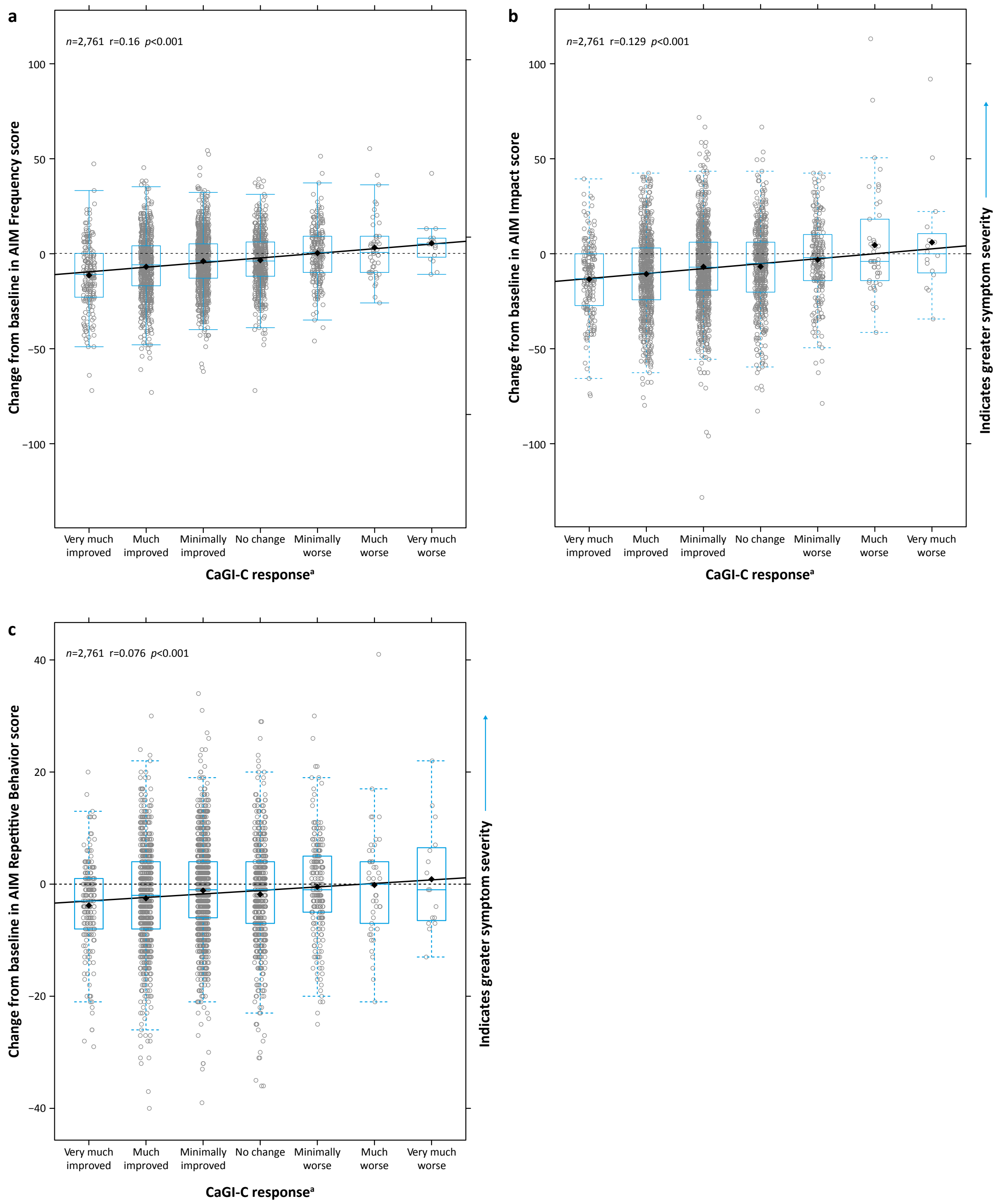

[a]Response to question 'Please indicate how much change your child has experienced between 12 months ago and today in his/her overall autism.'

Note. Possible score ranges for AIM frequency and impact dimensions are 41–205. Possible score range for AIM repetitive behavior domain is 16–80.

Abbreviations: *AIM* Autism Impact Measure, *CaGI-C* caregiver global impressions of change survey

Fig. S2 Change in AIM communication domain score (a), atypical behavior domain score (b), social reciprocity domain score (c), and peer interaction score (d) versus the primary anchor (overall caregiver impression of change)

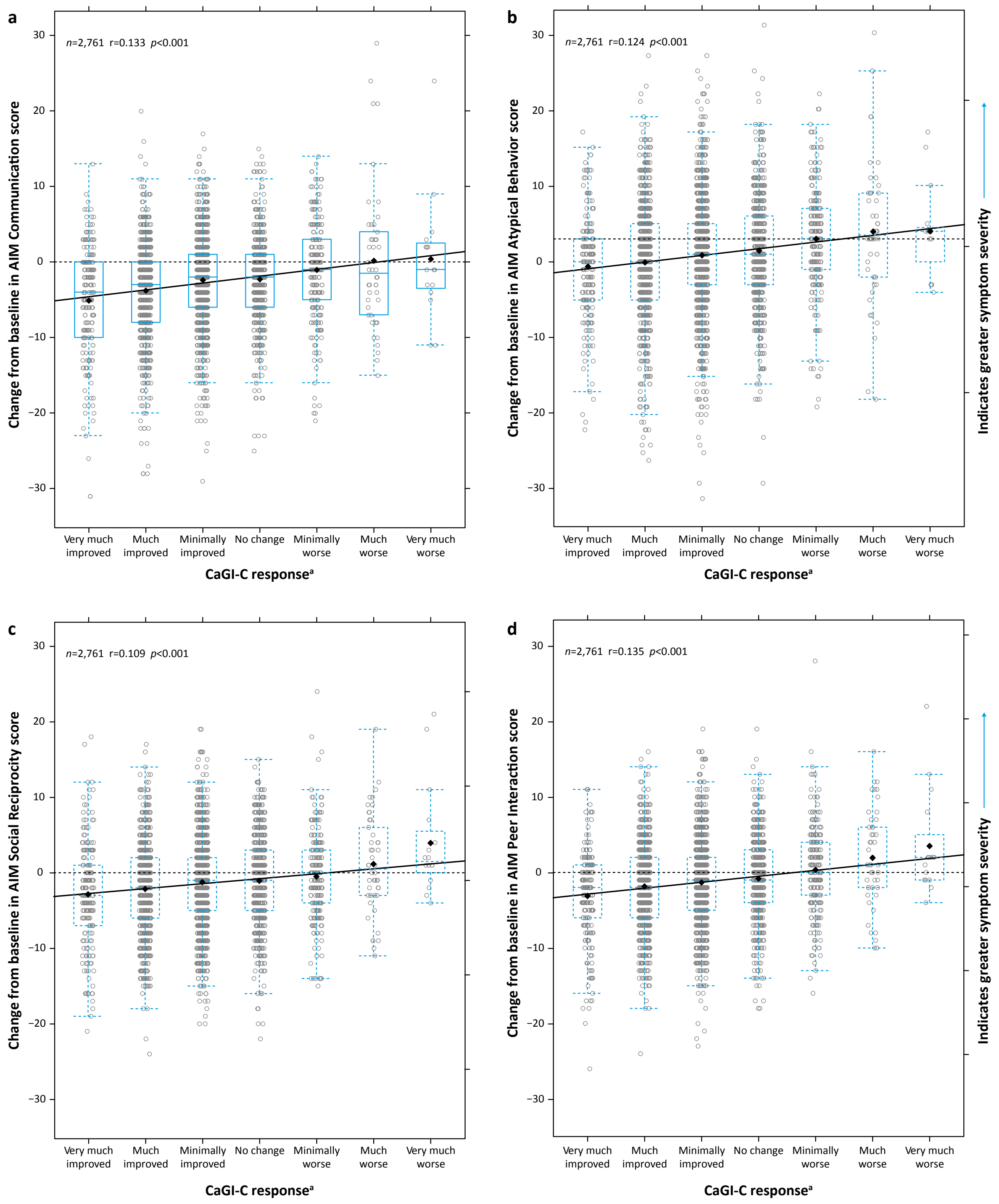

[a]Response to question ‘Please indicate how much change your child has experienced between 12 months ago and today in his/her overall autism.’

Note. Possible score ranges for AIM domains are 12–60 for communication, 12–60 for atypical behavior, 10–50 for social reciprocity, and 8–40 for peer interaction.

Abbreviations: *AIM* Autism Impact Measure, *CaGI-C* caregiver global impressions of change survey

Fig. S3 Change in AIM communication domain score versus the communication secondary anchor (overall caregiver impression of change)

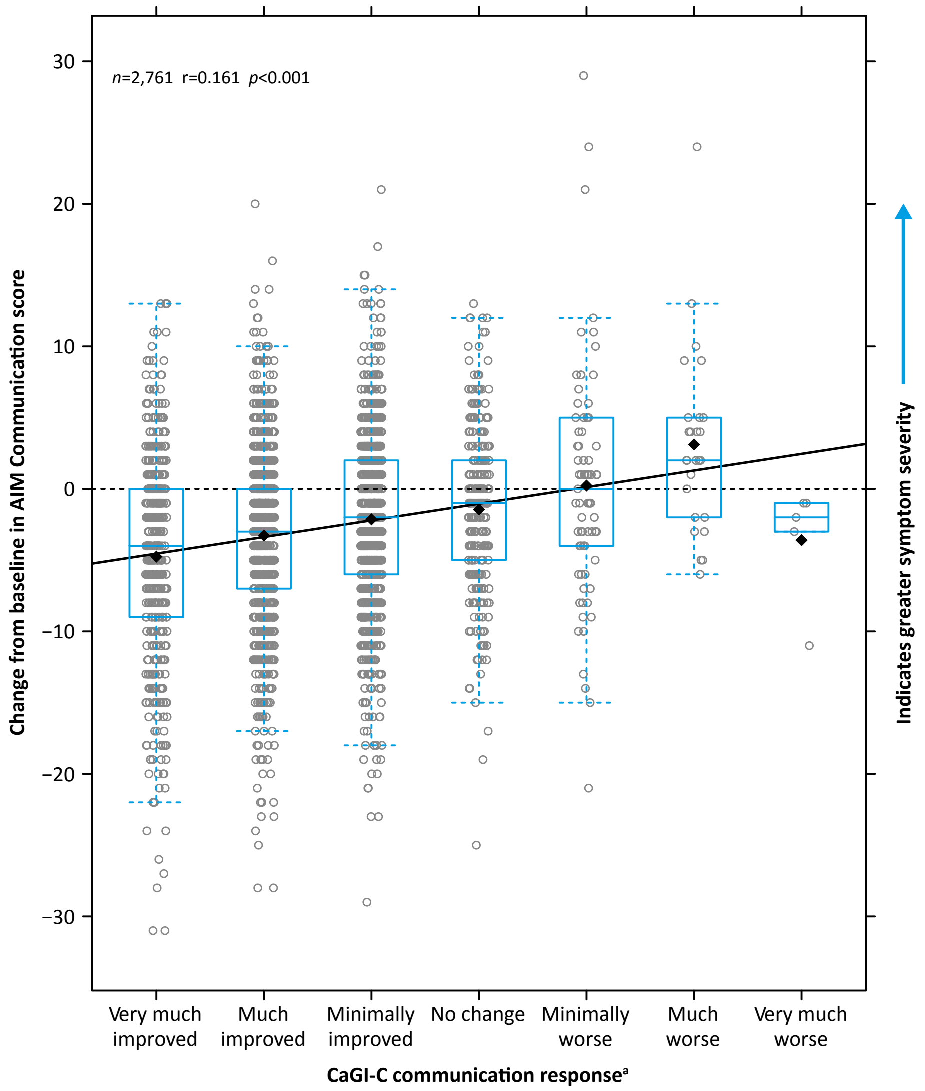

[a]Response to question ‘Please indicate how much change your child has experienced between 12 months ago and today in his/her communication with other people (e.g., speaking, listening and paying attention, understanding emotions of others, writing).’

Note. Possible score ranges for AIM communication domain is 12–60.

Abbreviations: *AIM* Autism Impact Measure, *CaGI-C* caregiver global impressions of change survey

Fig. S4 Change in AIM social reciprocity domain score (a) and peer interaction domain score (b) versus the social interaction secondary anchor (overall caregiver impression of change)

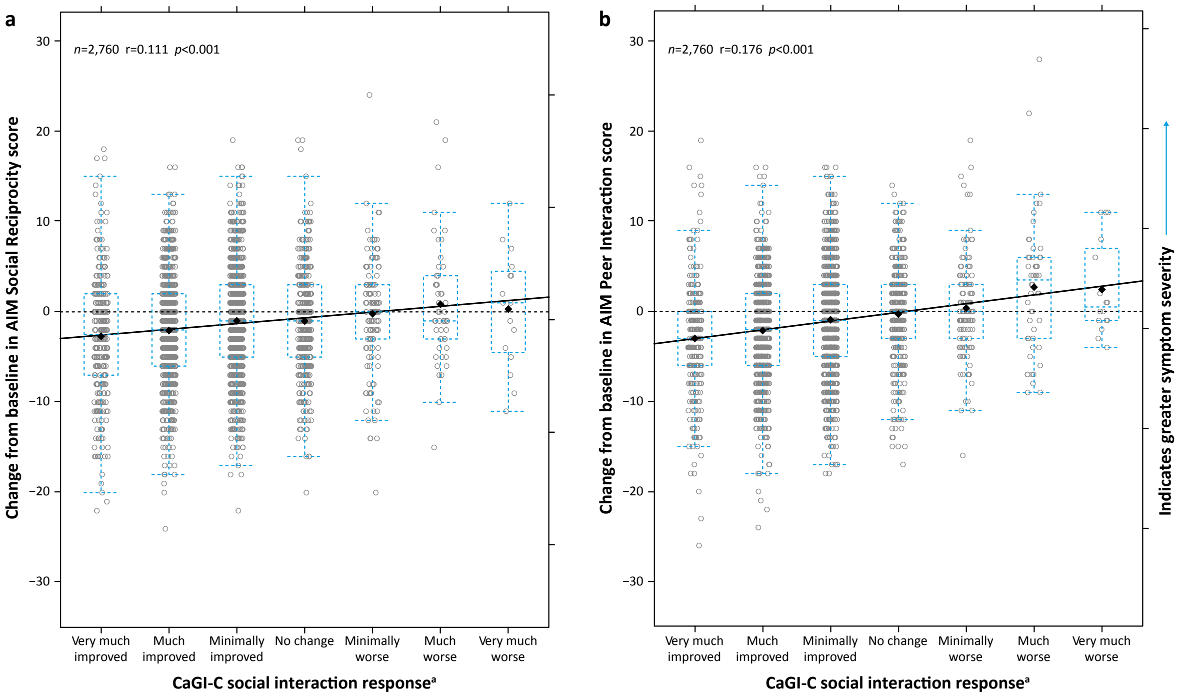

[a]Response to question ‘Please indicate how much change your child has experienced between 12 months ago and today in his/her social interaction with other people (e.g., getting along with others, socializing, coping in group situations).’

Note. Possible score ranges for AIM domains are 10–50 for social reciprocity and 8–40 for peer interaction.

Abbreviations: *AIM* Autism Impact Measure, *CaGI-C* caregiver global impressions of change survey

Fig. S5 AIM domain scores versus caregiver impression of severity

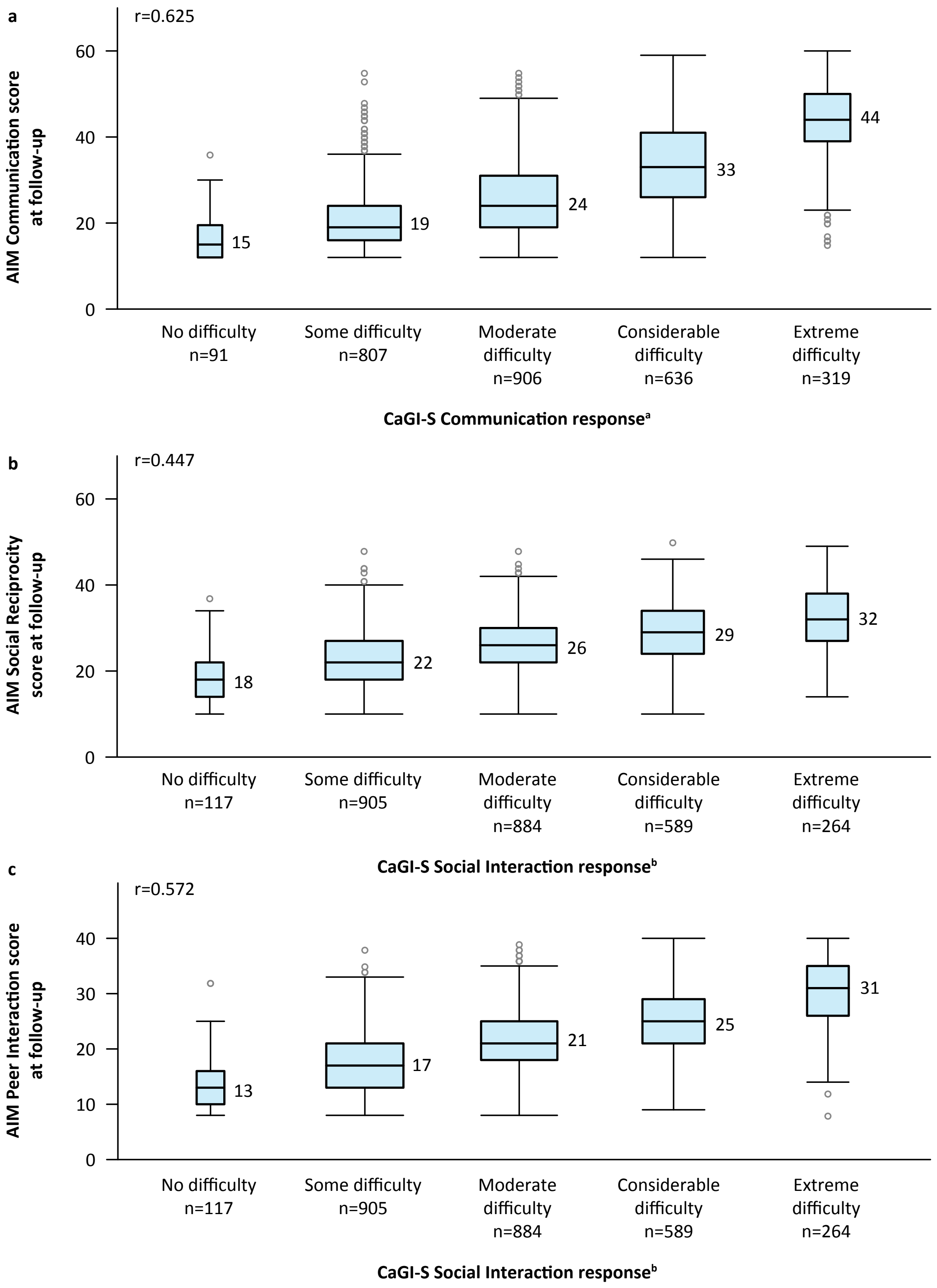

[a]Response to question 'Please tell us how much difficulty your child has experienced in the past two weeks in his/her communication with other people (e.g., speaking, listening and paying attention, understanding emotions of others, writing).'

[b]Response to question 'Please tell us how much difficulty your child has experienced in the past two weeks in his/her social interaction with other people (e.g., getting along with others, socializing, coping in group situations).'

Note. Possible score ranges for AIM domains are 12–60 for communication, 10–50 for social reciprocity, and 8–40 for peer interaction.

Abbreviations: *AIM* Autism Impact Measure, *CaGI-S* caregiver global impressions of severity survey

Fig. S6 Primary and secondary anchor-based within-person meaningful percent change thresholds for improvement and deterioration

Percent change[a] in AIM score

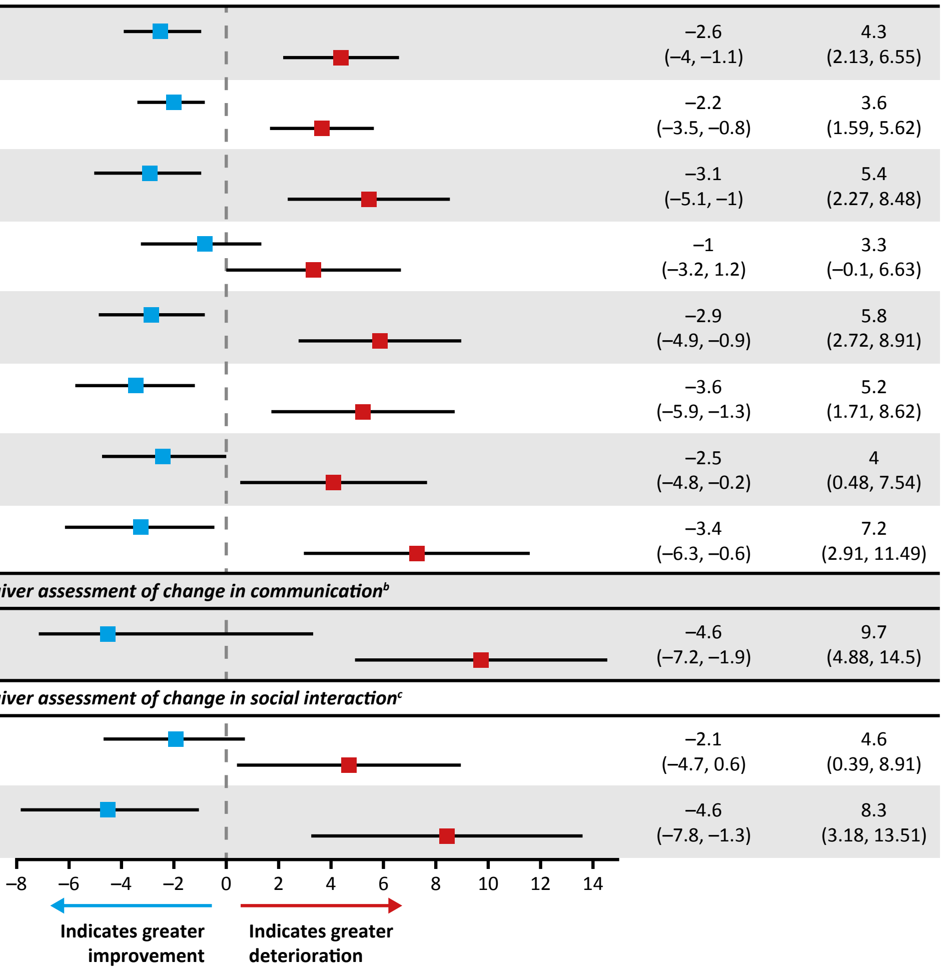

AIM domain	Meaningful change thresholds: Improvement (95% CI)	Meaningful change thresholds: Deterioration (95% CI)
Primary anchor: caregiver assessment of change in overall ASD[b]		
Total AIM	−2.6 (−4, −1.1)	4.3 (2.13, 6.55)
Total AIM Frequency	−2.2 (−3.5, −0.8)	3.6 (1.59, 5.62)
Total AIM Impact	−3.1 (−5.1, −1)	5.4 (2.27, 8.48)
Repetitive Behavior	−1 (−3.2, 1.2)	3.3 (−0.1, 6.63)
Communication	−2.9 (−4.9, −0.9)	5.8 (2.72, 8.91)
Atypical Behavior	−3.6 (−5.9, −1.3)	5.2 (1.71, 8.62)
Social Reciprocity	−2.5 (−4.8, −0.2)	4 (0.48, 7.54)
Peer Interaction	−3.4 (−6.3, −0.6)	7.2 (2.91, 11.49)
Secondary anchor: caregiver assessment of change in communication[b]		
Communication	−4.6 (−7.2, −1.9)	9.7 (4.88, 14.5)
Secondary anchor: caregiver assessment of change in social interaction[c]		
Social Reciprocity	−2.1 (−4.7, 0.6)	4.6 (0.39, 8.91)
Peer Interaction	−4.6 (−7.8, −1.3)	8.3 (3.18, 13.51)

[a]Percent change relative to the baseline value

[b]*N*=2,761

[c]*n*=2,760

Abbreviations: *AIM* Autism Impact Measure, *ASD* autism spectrum disorder, *CI* confidence interval

Fig. S7 Primary and secondary anchor-based within-person meaningful change thresholds for much improvement and much deterioration

AIM domain	Meaningful change thresholds: Much improvement (95% CI)	Meaningful change thresholds: Much deterioration (95% CI)
Primary anchor: caregiver assessment of change in overall ASD[a]		
Total AIM	−9.30 (−11.89, −6.71)	18.28 (10.05, 26.51)
Total AIM Frequency	−4.52 (−5.74, −3.30)	7.06 (3.18, 10.94)
Total AIM Impact	−4.78 (−6.45, −3.10)	11.22 (5.90, 16.54)
Repetitive Behavior	−1.53 (−2.25, −0.80)	1.38 (−0.92, 3.67)
Communication	−1.86 (−2.37, −1.35)	2.43 (0.80, 4.05)
Atypical Behavior	−1.48 (−2.09, −0.88)	2.67 (0.76, 4.59)
Social Reciprocity	−1.19 (−1.67, −0.71)	2.97 (1.45, 4.50)
Peer Interaction	−1.14 (−1.61, −0.68)	3.31 (1.84, 4.78)
Secondary anchor: caregiver assessment of change in communication[a]		
Communication	−1.87 (−2.35, −1.39)	3.90 (1.62, 6.18)
Secondary anchor: caregiver assessment of change in social interaction[b]		
Social Reciprocity	−1.29 (−1.75, −0.82)	1.65 (0.16, 3.14)
Peer Interaction	−1.67 (−2.11, −1.23)	3.30 (1.88, 4.72)

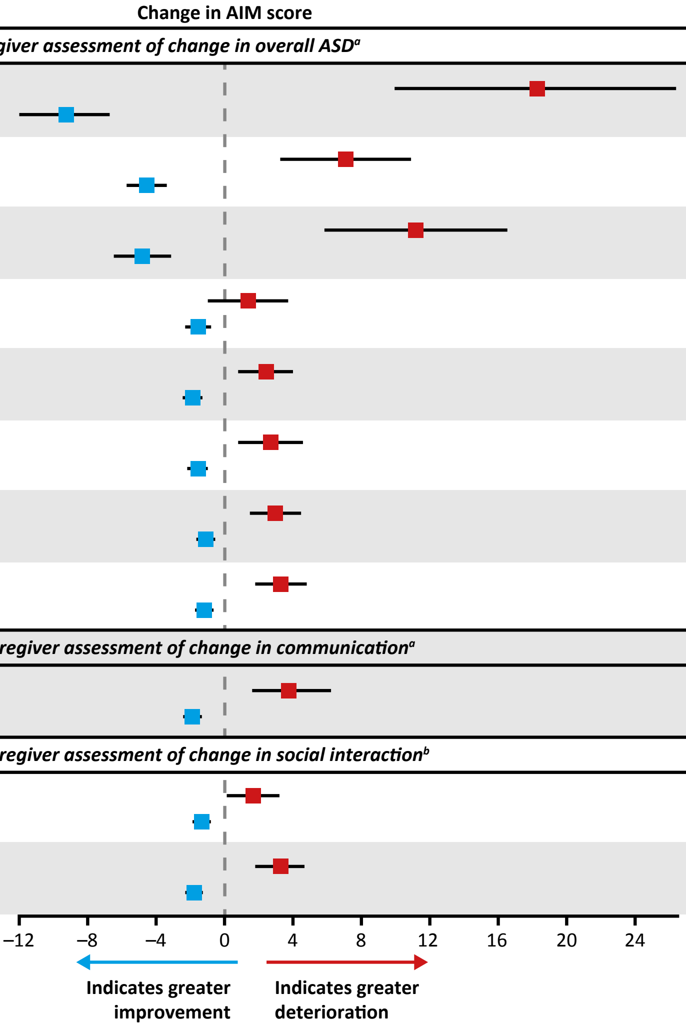

[a]*N*=2,761
[b]*n*=2,760
Abbreviations: *AIM* Autism Impact Measure, *ASD* autism spectrum disorder, *CI* confidence interval

Fig. S8 Primary and secondary anchor-based within-person meaningful percent change thresholds for much improvement and much deterioration

AIM domain	Percent change[a] in AIM score	Meaningful change thresholds: Much improvement (95% CI)	Meaningful change thresholds: Much deterioration (95% CI)
Primary anchor: caregiver assessment of change in overall disease severity[b]			
Total AIM		−4.80 (−6.00, −3.50)	8.00 (4.13, 11.80)
Total AIM Frequency		−4.00 (−5.10, −2.90)	5.40 (1.92, 8.92)
Total AIM Impact		−5.50 (−7.20, −3.80)	11.70 (6.29, 17.07)
Repetitive Behavior		−3.70 (−5.50, −1.90)	2.80 (−3.01, 8.70)
Communication		−6.20 (−7.90, −4.50)	9.80 (4.41, 15.15)
Atypical Behavior		−4.80 (−6.70, −2.90)	8.10 (2.05, 14.10)
Social Reciprocity		−4.60 (−6.50, −2.70)	11.20 (5.05, 17.33)
Peer Interaction		−5.20 (−7.60, −2.90)	12.70 (5.18, 20.16)
Secondary anchor: caregiver assessment of change in communication[b]			
Communication		−6.20 (−7.80, −4.60)	12.70 (5.19, 20.27)
Secondary anchor: Caregiver assessment of change in social interaction[c]			
Social Reciprocity		−4.50 (−6.30, −2.60)	7.20 (1.25, 13.21)
Peer Interaction		−7.50 (−9.70, −5.30)	16.30 (9.11, 23.58)

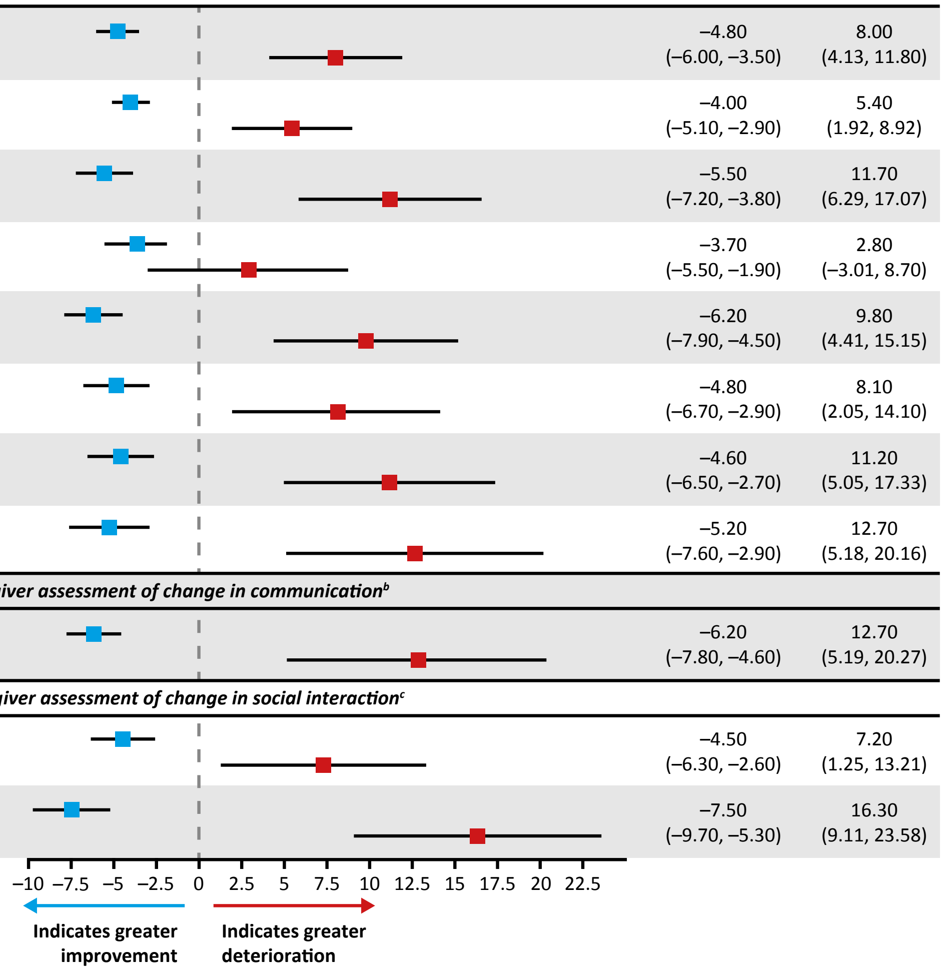

[a]Percent change relative to the baseline value

[b]*N*=2,761

[c]*n*=2,760

Abbreviations: *AIM* Autism Impact Measure, *ASD* autism spectrum disorder, *CI* confidence interval
